# Forest Cover and Altitude Are Key to the Occurrence of Black‐Fronted Titi Monkeys (*Callicebus nigrifrons*) in the Brazilian Atlantic Forest

**DOI:** 10.1002/ajp.70079

**Published:** 2025-09-19

**Authors:** Aron Silvarolli Fernandes, Lisieux Fuzessy, Carla C. Gestich, Felipe Martello, Milton Cezar Ribeiro

**Affiliations:** ^1^ Institute of Biosciences São Paulo State University (UNESP) Rio Claro São Paulo Brazil; ^2^ Department of Genetics and Evolution Federal University of São Carlos (UFSCar) São Carlos São Paulo Brazil; ^3^ School of Geography and Environment University of Oxford Oxford Oxfordshire United Kingdom; ^4^ Environmental Studies Center São Paulo State University (UNESP) Rio Claro São Paulo Brazil

**Keywords:** conservation biology, landscape ecology, platyrrhine, tropical ecology

## Abstract

Human activities are major threats to biodiversity, particularly for arboreal, forest‐specialist species such as platyrrhine primates. Habitat loss and declines in vegetation quality negatively affect species' persistence in disturbed and human‐modified landscapes. In this study, we evaluated the influence of landscape structure (forest cover and functional connectivity), vegetation quality and structure (EVI and canopy height), fire disturbance history (% burned area), and altitude on the occurrence of black‐fronted titi monkeys (*Callicebus nigrifrons*) in the Cantareira‐Mantiqueira Corridor, a region within the Atlantic Forest biodiversity hotspot. We conducted playback surveys at 72 sites to detect the presence of the species. Using model selection approaches, we assessed the relative importance of environmental predictors on the species' occurrence. The best‐supported model included forest cover (within a 250 m radius) and altitude, indicating that greater forest cover at higher elevations best explains the presence of black‐fronted titi monkeys. Variables related to functional connectivity, vegetation quality and structure and fire disturbance history had secondary importance. Our findings underscore the critical importance of preserving and restoring forested areas, particularly mid‐elevation ranges (500–1200 m). These regions face the most severe degradation, posing a significant threat to black‐fronted titi monkeys, a species currently listed as Near Threatened on the IUCN Red List. Our results align with previous studies showing positive associations between forest cover and the occurrence of congeneric species, reinforcing the urgent need for targeted conservation actions in increasingly degraded habitats.

## Graphical abstract

Conceptual diagram illustrating how altitude and forest cover jointly influence the probability of primate occurrence. Higher altitudes and greater forest cover are associated with increased occurrence probability. Icons on the axes represent gradients of altitude (left) and forest cover (bottom), while primate silhouettes depict different probabilities of occurrence along the gradient.

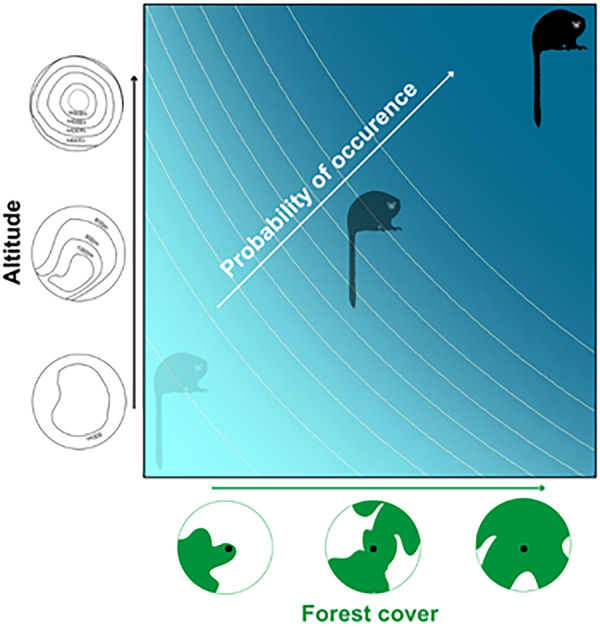

## 1 Introduction

Human activities, such as urbanization and the conversion of natural habitats into agricultural and livestock areas, significantly alter native landscapes and reduce the quantity and quality of vital resources for biodiversity maintenance (Johnson et al. 2017). Habitat loss is among the most severe consequences of these activities, affecting ecosystems (Martensen et al. 2012; Pardini et al. 2009) and various ecological responses, including animal movement (Fahrig 2007), interspecific interactions (Polis et al. 2004), and ecological functions (Lovett et al. 2005). The ability of species to access resources and disperse across fragmented landscapes—known as functional connectivity—is particularly compromised when habitat loss impairs potential movement corridors (Urban and Keitt 2001; Martensen et al. 2008). Consequently, habitat loss can reduce species occurrence and functional diversity (Pereira et al. 2022; Benchimol and Peres 2013) and negatively affect critical ecological processes due to species loss (Cazetta and Fahrig 2022).

Tropical forests, particularly in the Americas, are among the most threatened habitats due to deforestation and degradation (Newbold et al. 2016; Caetano‐Andrade et al. 2020; Roberts et al. 2021). Many animal species are confined to human‐modified landscapes (Magioli et al. 2019), where remaining forest areas are often surrounded by pastures, agricultural land, silviculture, and urban areas (Watling et al. 2011; Galán‐Acedo et al. 2019a; Hendershot et al. 2020). For forest‐dependent animals, population persistence in such human‐modified landscapes depend not only on habitat quantity but also on functional connectivity and access to complementary resources across the matrix (Henle et al. 2004). This is the case for nonhuman primates, of which approximately 94% are forest‐dependent (Galán‐Acedo et al. 2019b), and around 66% are currently at risk of extinction according to the IUCN, primarily due to habitat loss from farming and logging (Estrada et al. 2017). Despite this, the effects of altered landscapes on nonhuman primates remain poorly understood.

Primate persistence in a landscape is influenced by multiple factors, including landscape structure, habitat quality, resource availability, and environmental conditions. These factors shape the distribution and nutritional value of food resources (Wieczkowski 2004), alter microclimates (Estrada and Garber 2022), and change vegetation structure (Arroyo‐Rodríguez et al. 2020). Recent studies show that habitat quality parameters, such as canopy cover, tree height, and vegetation indices (e.g., Enhanced Vegetation Index, EVI), can predict primate occurrence, complementing spatial metrics and balancing the effects of reduced habitat area (Hamard et al. 2010; Costa‐Araújo et al. 2021).

Abiotic factors, such as climate, topography, and soil properties, also strongly influence primate distributions (Carvalho et al. 2019; Pebsworth et al. 2019). For instance, altitudinal gradients create distinct ecological conditions that affect floristic composition, vegetation structure, and primary productivity—factors that directly affect primate communities (Cardoso et al. 2019; Rezende et al. 2015). Changes in plant community composition along altitudinal gradients alter food resource availability (Brown 2001), with colder temperatures at higher elevations disproportionately impact folivorous species (Beaudrot and Marshall 2019). In some regions of the Atlantic Forest, higher elevations (> 920 m) exhibit greater forest cover (Silva et al. 2007) and enhanced canopy connectivity, due to these areas having lost a smaller proportion of their original forest cover (Tabarelli et al. 2010). These conditions can support primate persistence by (1) providing continuous arboreal pathways (de Guinea et al. 2019), (2) buffering thermal extremes (Korstjens et al. 2018), and (3) sustaining resource availability via asynchronous phenological patterns across elevations (Morellato et al. 2000). These interactions highlight how abiotic factors mediate primate distributions both directly, via physiological constraints, and indirectly, through their influence on habitat quality.

Additionally, fire history can affect primate occurrence in human‐modified landscapes because in agricultural landscapes, fire is widely used to clear land, renew pastures, and control pests (Jacques 2003; Goldammer 2016). However, these fires often spread to nearby forests, harming native vegetation and wildlife. The Atlantic Forest is particularly vulnerable, as fires disrupt ecological processes and threaten species that are unadapted to such disturbances (Hardesty et al. 2005). In this biome, fires can drastically alter mammalian communities by reducing population abundance. For instance, Canale et al. (2012) report that medium‐ to large‐bodied species tend to decline in both forest interiors and edges. Fire hotspots can affect primates directly or indirectly, depending on their mobility (Frizzo et al. 2011; Cochrane 2003). Therefore, fire disturbance history should be considered when assessing the occurrence patterns of forest‐dependent species in altered landscapes.

Vegetation quality, under the resource availability hypothesis, is another key factor that can influence primate occurrence in forest fragments. *Callicebus nigrifrons*, for instance, despite exhibiting some dietary flexibility, prefers high‐nutritional‐value food items (Nagy‐Reis and Setz 2017), which are usually more abundant in structurally complex, high‐quality habitats. As mid‐stratum dwellers, these primates are likely sensitive to forest structure, where factors such as tree height and vertical complexity may constrain their occurrence (Trevelin et al. 2007). Thus, older, well‐structured forests with mature canopy architecture may provide essential conditions for *Callicebus* persistence.

Titi monkeys (*Callicebus* spp.) inhabit densely populated and heavily exploited regions of Brazil, formerly dominated by the Atlantic Forest—one of the world's most threatened biodiversity hotspots (Jerusalinsky et al. 2020; Myers et al. 2000). The black‐fronted titi monkey (*Callicebus nigrifrons*), endemic to this biome, is an excellent model for evaluating primate persistence across landscape change gradients. Currently listed as Near Threatened by the IUCN Red List, the species is undergoing a population decline (Jerusalinsky et al. 2020). This small primate (adult body mass < 1.5 kg) lives in family groups of two to six individuals and feeds mainly on fruits, with some leaf consumption (Nagy‐Reis and Setz 2017). The species also exhibits a complex vocal repertoire, with duets composed of multiple alternating calls (Müller and Anzenberger 2002), which makes it highly responsive to playback surveys, similar to other congeners (Gestich et al. 2019).

Given that habitat loss is a primary driver of population declines in platyrrhine primates (see Estrada et al. 2017), we propose that integrating variables related to landscape structure, abiotic conditions, vegetation quality and structure, and fire disturbance history will provide more accurate estimates of *Callicebus nigrifrons* occurrence in human‐modified landscapes. Thus, this study evaluates the effects of these factors on the occurrence of black‐fronted titi monkeys.

We tested four nonexclusive hypotheses to evaluate primate occurrence patterns. First, the resource availability hypothesis predicts that forest cover, vegetation quality and structure—measured as forest cover percentage, EVI, and canopy height—positively influence occurrence by increasing food and shelter availability (Hanya and Chapman 2012). Accordingly, we predicted that black‐fronted titi monkey occurrence would be positively associated with forest cover and vegetation quality and structure. Second, the disturbance sensitivity hypothesis suggests that fire history negatively affects occurrence due to the species' dependence on stable canopy structure (Godoy et al. 2025). We therefore predicted a negative association between fire disturbance and species occurrence. Third, the altitude refuge hypothesis states that higher elevations (> 920 m) enhance species persistence, likely due to reduced human pressure and improved vegetation quality (Silva et al. 2007; Amorim et al. 2009). Based on this, we predicted a positive association between species occurrence and elevation. Fourth, the landscape connectivity hypothesis proposes that functional connectivity promotes occurrence by facilitating movement and gene flow (Magioli et al. 2016). We thus predicted a positive association between functional connectivity and species occurrence. Given the species' strong reliance on forest habitats, we expected landscape structure—particularly forest cover—to be the main factor influencing the distribution of the black‐fronted titi monkey, surpassing the effects of vegetation quality and structure, fire disturbance history, and altitude.

## 2 Methods

### 2.1 Ethics Statement

This project was approved by the Scientific Council of the Environmental Research Institute, in accordance with Deliberation CC/IPA number 001/2022 (Digital process SIMA.058303/2021‐91). Our research adhered to the ethical principles for the treatment of primates established by the American Society of Primatologists.

### 2.2 Study Area

**Figure 1:**
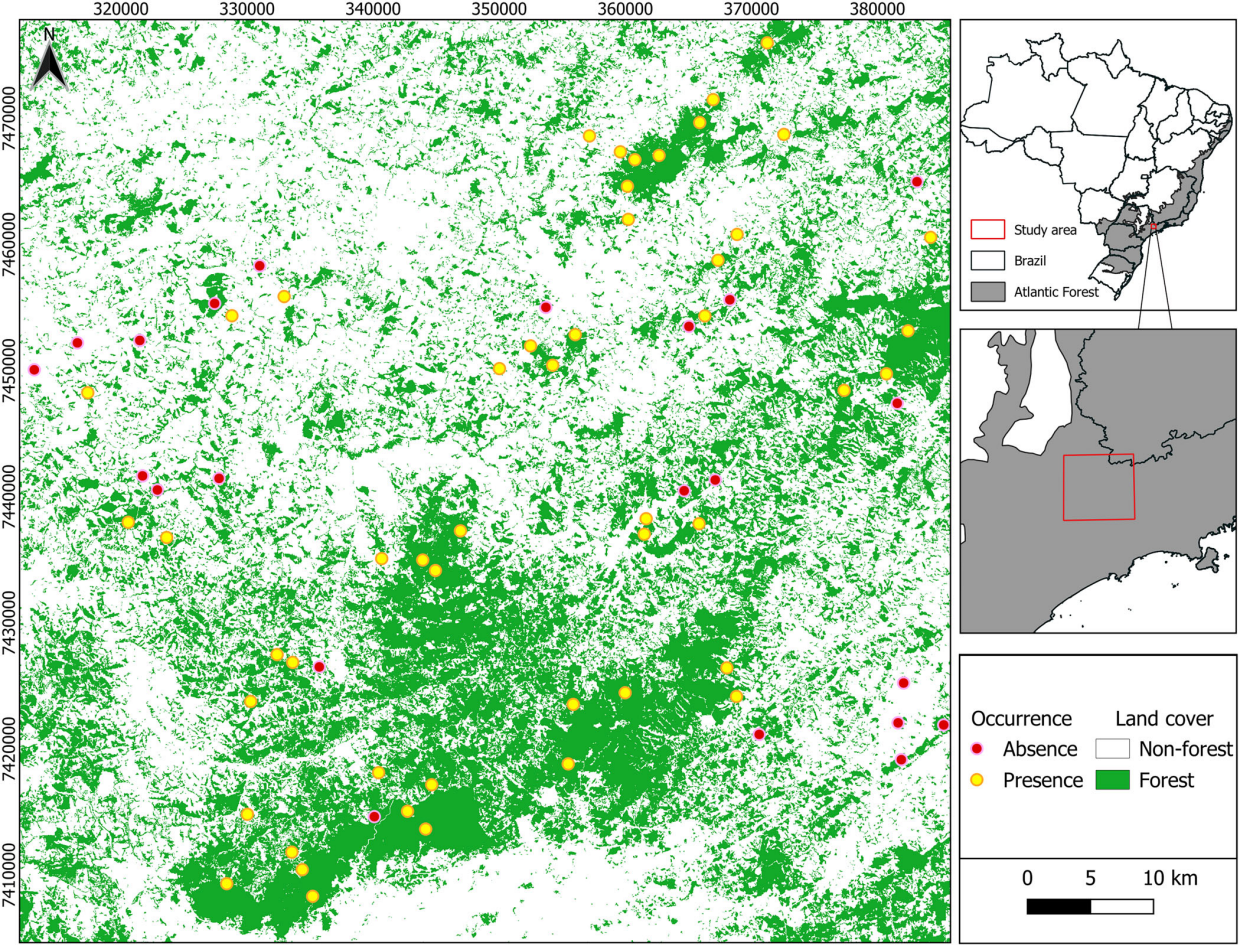
Location of sampling sites for black‐fronted titi monkey (*Callicebus nigrifrons*) surveys and corresponding land cover in the Cantareira‐Mantiqueira Corridor, in the states of São Paulo and Minas Gerais states, Brazil. Yellow dots indicate presence, and pink dots indicate absence.

The study was conducted in the Cantareira‐Mantiqueira Corridor (CCM), located in the Atlantic Forest biome, in the northwestern region of São Paulo state and the southern portion of Minas Gerais state. This region has hosted a Long‐Term Ecological Research (LTER‐CCM) project (Figure 1). The CCM covers approximately 727,000 ha across 42 municipalities and encompasses two of the largest continuous masses of native vegetation in the Atlantic Forest: Serra da Cantareira and Serra da Mantiqueira (Rodrigues 2008). The landscape consists of a mosaic of protected areas, forest patches of varying sizes, pastures, forestry, agricultural lands, urban areas, and regenerating zones. Native vegetation constitutes 35% of the total area, mostly composed of secondary vegetation that regenerated after extensive coffee cultivation until the late 19th century. The remaining land is dominated by anthropogenic matrices: agriculture and pasture (43%), urban areas (11%), and eucalyptus plantations (6%) (Montagnana 2018).

The region has a mesothermal and humid climate, with rainy summers and dry winters (Cwa under the Köppen climate classification). Mean temperatures peak at ≈23°C between January and February and drop to ≈16.6°C between June and July. The region is sub‐mountainous to mountainous, with altitudes ranging from 600 to 2000 m. Annual rainfall mean ≈1500 mm (Montes 2005).

### 2.3 Study Design

We selected 72 sites (Figure 1; see Supporting Information S1: Tables S1 and S2), each separated by a minimum distance of 1 km to minimize the likelihood of repeatedly detecting the same primate group. Adopting a landscape‐scale study approach (Arroyo‐Rodríguez and Fahrig 2014), we strategically distributed the sites across a forest cover gradient to capture the full range of landscape variability and ensure comprehensive representation of the landscape metrics analyzed.

### 2.4 Primate Surveys

We recorded black‐fronted titi monkey occurrences in forest areas using playback stimuli. Surveys were conducted from March 2022 to November 2023, totaling 59 field days and 133 visits to 72 sampling sites. Some sites were revisited to confirm presence or absence. Playback sessions began about 1 h after sunrise (~06:00 AM) and lasted 5 h, coinciding with the peak vocal activity of *C. nigrifrons* (Caselli and Setz 2007). Each session lasted 9 min and comprised two playback periods separated by silent intervals. Sampling points were positioned at least 100 m from forest edges to minimize edge effects while maintaining accessibility. Sessions were suspended during adverse weather conditions, such as strong winds and rain.

We used a MiniVox portable speaker (Anchor, Carlsbad, CA) connected to an audio player. The speaker had a frequency response of 100–15,000 Hz, an output power of 30 W, and a maximum sound pressure level of 109 dB, mimicking the species natural vocalizations and enabling detection of responses up to 350 m (Gestich et al. 2017). Volume was calibrated to resemble natural calls as perceived by human hearing and was kept consistent throughout the study.

Following the procedures of Gestich et al. (2017), each playback session consisted of 1 min of black‐fronted titi monkey vocalization playback, followed by 2 min of silence, then a second 1‐min playback and a 5‐min observation period. If the species was not detected, the site was revisited after at least 15 days, with intervals of 15–170 days. Return visits were spaced to avoid habituation to the playback stimulus and to confirm species absence. If the species was detected on the first or second visit, no further surveys were performed at that site.

Species presence was confirmed through vocal responses or direct sightings. A site was classified as a presence point only if vocalizations originated from the area containing the sampling point. To ensure this, we triangulated the vocalization directions and cross‐referenced them with mapped forest boundaries. In continuous forest areas, we confirmed presence only when vocalizations occurred near the observer. In addition to playback detections, we also determined species presence through visual observations and records from the LTER‐CCM team.

### 2.5 Landscape Analysis

We calculated landscape metrics using land cover data from MapBiomas Collection BETA (MapBiomas 2023a), with a 10‐m pixel resolution. We categorized land cover into two categories: “Non‐Forest” and “Forest.” The “Forest” category included areas with tree vegetation and natural regeneration older than 10 years. Land cover data correspond to the year 2022.

To determine the best spatial scale for explaining black‐fronted titi monkey occurrence, we conducted a multi‐scale analysis following established methods (Jackson and Fahrig 2015; Gestich et al. 2019). From each point, we calculated the percentage of forest cover within circular buffers of 250, 500, 750, and 1000 m radii. These distances were selected to ensure scale independence and reflect ecologically meaningful distances, particularly considering the smallest documented home range of the species (Nagy‐Reis and Setz 2017). We then performed linear regressions between species occurrence and forest cover at each scale and selected the optimal scale based on the highest coefficient of determination (R²; see Supporting Information S1:Table S3). Analyses were conducted in R version 4.2.3 (R Core Team 2024) using the *landscapemetrics* package (Hesselbarth et al. 2019).

To assess functional connectivity, we adopted the graph theory approach developed by Martensen et al. (2012), incorporating species‐specific movement capabilities, including corridor use and matrix crossing between patches (Urban and Keitt 2001; Martensen et al. 2008). We applied a 100‐m gap‐crossing threshold, merging adjacent forest fragments separated by ≤ 100 m to reflect the known ability of *Callicebus* species to traverse pasture‐dominated matrices (Souza‐Alves et al. 2019). We calculated the total forest area connected by corridors, excluding the occurrence forest patch but including both the corridors and connected forest patches. This metric also encompassed any total forest area accessible from the occurrence forest patch by crossing a 100‐meter gap. Connectivity metrics were calculated using the *LSMetrics* package in GRASS GIS (https://github.com/LEEClab/LS_METRICS).

### 2.6 Local Variables

#### 2.6.1 Fire Disturbance History and Altitude

Fire disturbance history and EVI were treated as local environmental variables because they reflect conditions that directly affect resource availability, vegetation structure, and microclimate at the scale experienced by the monkeys during daily activities. We assessed fire disturbance history using data from MapBiomas Fire Collection 2.0, with a 30‐meter pixel resolution (MapBiomas 2023b). For each sampling point, we calculated the total burned area within a 250‐meter buffer from 2003 to 2022 and averaged this value over the 20‐year period using RStudio. We also calculated the mean altitude within a 250‐meter buffer using 30‐meter resolution digital elevation models (European Space Agency 2024). Ecological processes such as foraging, movement, and territory use in small‐bodied primates like *Callicebus nigrifrons* typically occur within a few hundred meters (Nagy‐Reis and Setz 2017), making the 250‐meter buffer biologically meaningful. Moreover, this buffer size reduced fine‐scale variability associated with individual pixels and ensured that estimates accurately represented the immediate surroundings of each sampling site, minimizing the influence of extreme local heterogeneity while preserving ecological relevance.

#### 2.6.2 Vegetation Quality and Structure

We estimated vegetation quality and structure using two structural metrics: the mean Enhanced Vegetation Index (EVI) and mean canopy height. These indicators provide complementary information on critical aspects of vegetation structure and habitat quality and capture key horizontal and vertical dimensions of habitat quality relevant to black‐fronted titi monkeys (Huete et al. 2002). EVI was used as a proxy for habitat productivity and complexity. We calculated mean EVI values within 250‐meter buffers around each sampling point using 10‐meter resolution Sentinel‐2 satellite images. Data were collected monthly from March 2022 to November 2023. Remote sensing‐derived vegetation quality proxies, such as EVI (Huete et al. 2002), can capture both structural and nutritional habitat attributes and are useful predictors of primate occurrence. EVI indirectly reflects vegetation quality and was designed for use in tropical forests (Huete et al. 2002). It remains robust under conditions of heavy aerosols and biomass burning (Miura et al. 1998) and correlates with indicators of gross primary production (e.g., leaf area index, LAI), chlorophyll content, and canopy density—key attributes of suitable habitats for arboreal primates, including black‐fronted titi monkeys (Huete et al. 2002; Ogaya et al. 2015). Higher EVI values typically reflect mature, old‐growth forests with complex structures, whereas lower values indicate early successional or disturbed forests (Cazzolla Gatti et al. 2015; Nyirambangutse et al. 2017).

Mean canopy height, the second metric, was used as a direct measure of vertical forest structure. We obtained mean canopy height values within 250‐meter buffers from a global canopy height map with 1‐meter resolution (Tolan et al. 2024). Taller canopies are associated with mature forests that provide abundant resources, complex vertical strata, and greater microclimate stability, whereas shorter canopies often reflect secondary or degraded forests (LIDAR 2002; Tolan et al. 2024). The Global Canopy Height Maps data set offers comprehensive insights into tree canopy heights worldwide, providing an overview of tree canopy presence and height for the analyzed period (2009–2020), with 80 percent of the data obtained from imagery acquired between 2018 and 2020 (Tolan et al. 2024).

### 2.7 Data Analysis

We used generalized linear models (GLMs) with a binomial distribution to assess the influence of landscape structure (i.e., forest amount and functional connectivity) and vegetation quality and structure, topography, and fire disturbance on the occurrence of black‐fronted titi monkeys. Species presence and absence (non‐detection) were coded as 1 and 0, respectively. We adopted a multi‐model averaging approach (Burnham and Anderson 2002) to evaluate both univariate and combined effects of the predictor variables.

To assess the relative importance of each predictor variable, we fitted a full model including all predictors and their combinations. This was implemented in R using the *glmulti* package (Calcagno and Mazancourt 2010), which performs automated model selection based on the Akaike Information Criterion (AIC). The set of candidate models included the following predictors: forest cover (%), functional connectivity, EVI, fire disturbance history, and altitude. This approach enabled us to identify the most influential predictors while accounting for potential multicollinearity and improving the robustness of our inferences regarding the drivers of primate occurrence.

All continuous predictors were standardized (mean = 0, SD = 1) before analysis to allow direct comparison of effect sizes across variables. Model performance was evaluated using AIC. Models with ΔAIC < 2 were considered equally plausible, and model weights (wAIC) were used for model averaging and to assess the relative importance of predictors.

Multicollinearity was assessed using Pearson correlation coefficients (threshold |r | > 0.7) and variance inflation factors (VIF > 3) for all predictors (see Supporting Information S1: Tables S4 and S5). A strong correlation was found only between forest cover and canopy height (*r* = 0.85), leading to the exclusion of models that included canopy height from further analyses. All remaining predictors showed acceptable levels of collinearity (VIF < 2, *r* < 0.7).

Model diagnostics were conducted using the *DHARMa* package (Hartig 2022), including residual inspection and tests for overdispersion, heteroscedasticity, and deviations from the assumed distribution. Although residuals slightly deviated from normality (Shapiro–Wilk *p* < 0.05), this is not an issue for GLMs, which assume a binomial—not normal—distribution of the response variable. No evidence of heteroscedasticity or systematic bias was detected.

To evaluate spatial autocorrelation in model residuals, we calculated global Moran's I using Monte Carlo simulations with the *pgirmess*, *spdep*, and *sp* packages. The result (Moran's I = 0.18, *p* = 0.058) indicated low and nonsignificant spatial autocorrelation, supporting the assumption of spatial independence among sampling sites (Wang et al. 2020).

## 3 Results

### 3.1 Black‐Fronted Titi Monkey Occurrence

Black‐fronted titi monkeys were detected at 50 of the 72 sampled sites (69.4%) and not detected at the remaining 22 sites (30.6%) (Figure 1; see Supporting Information S1: Tables S1 and S2).

### 3.2 Occurrence Modeling and Model Selection

**Figure 2:**
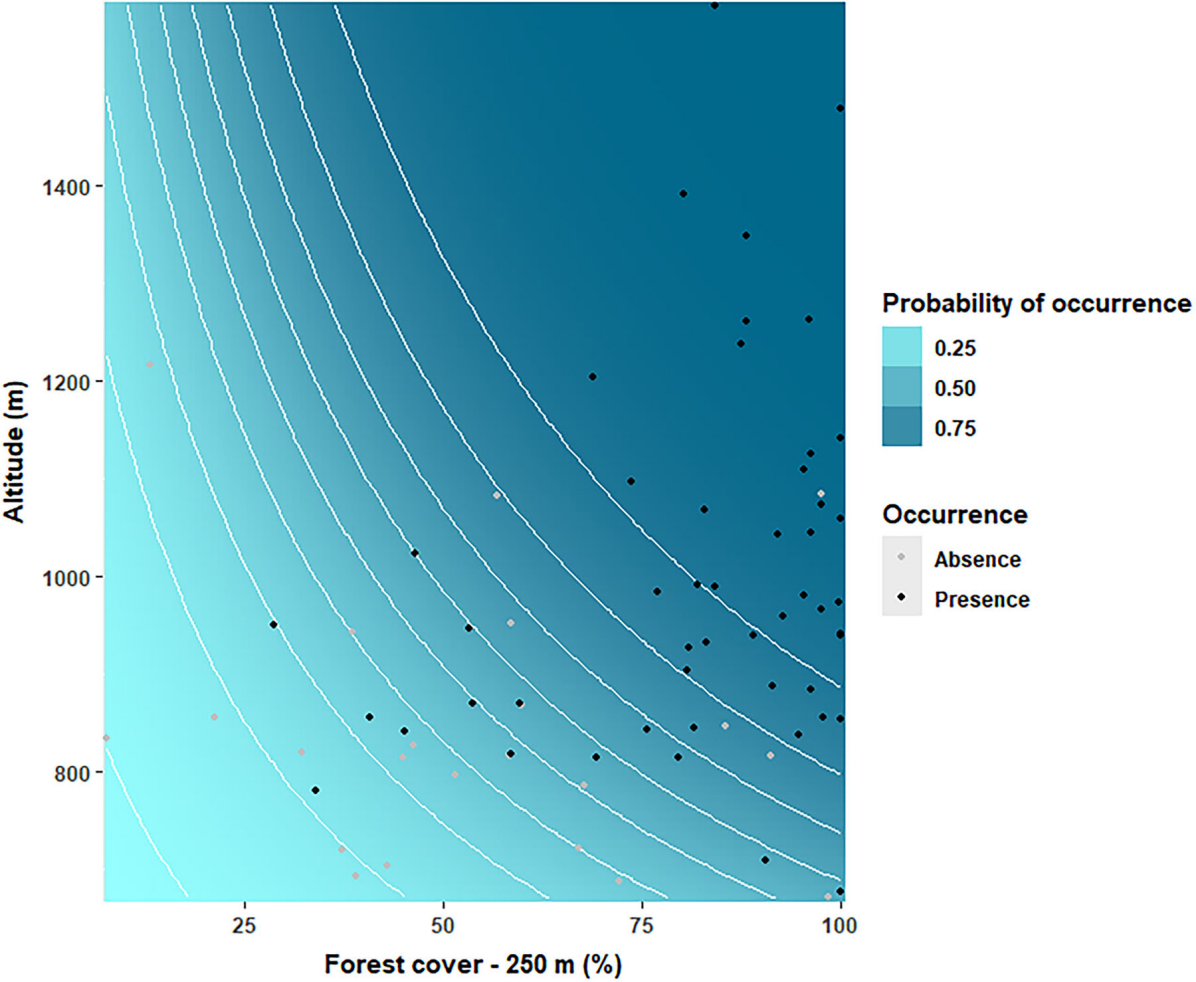
Response surface showing the probability of occurrence of black‐fronted titi monkeys (*Callicebus nigrifrons*) (z‐axis) as a function of forest cover within a 250‐m radius (x‐axis), and altitude (y‐axis). Black dots indicate presence, gray dots indicate absence, and white curved lines illustrate the relationship between altitude and forest cover.

We used a model selection framework to evaluate a comprehensive set of hypotheses by fitting all combinations of predictors in GLMs, including a full model with all variables (Table 1, see Supporting Information S1: Table S6 for the complete model). The most plausible model explaining the occurrence of black‐fronted titi monkeys included forest cover (within a 250 m buffer) and altitude. These variables contributed 98.4% and 92%, respectively, to our model (see Supporting Information S1: Table S7), with both showing positive associations with species occurrence. This suggests that habitat extent and topographic variation are key drivers of black‐fronted titi monkey distribution (Figure 2; see Supporting Information S1: Tables S6 and S7 for full model outputs).

**Table 1:** Akaike information criterion (AIC) values for the relative contribution of the highest‐ranked models explaining the occurrence of black‐fronted titi monkeys (*Callicebus nigrifrons*) in fragmented landscapes in the Cantareira‐Mantiqueira Corridor.

Model	AIC	ΔAIC	Weight
Forest cover + altitude	67.16	0.0	0.37
Forest cover + altitude + fire disturbance history	69.18	2.02	0.13
Forest cover + altitude + EVI	69.26	2.10	0.13
Forest cover + altitude + functional connectivity	69.39	2.23	0.12
Forest cover + altitude + fire disturbance history + EVI	71.39	4.23	0.04
Forest cover + altitude + functional connectivity + fire disturbance history	71.49	4.33	0.04
Forest cover + altitude + functional connectivity + EVI	71.51	4.35	0.04
Forest cover	72.64	5.48	0.02
Forest cover + altitude + functional connectivity + fire disturbance history + EVI	73.76	6.60	0.01
Forest cover + fire disturbance history	73.78	6.62	0.01
Forest cover + EVI	73.92	6.76	0.01
Forest cover + functional connectivity	74.44	7.28	0.01
Forest cover + EVI + fire disturbance history	75.37	8.21	< 0.01
Forest cover + functional connectivity + EVI	75.53	8.37	< 0.01
Functional connectivity + EVI + altitude	75.78	8.62	< 0.01
Forest cover + functional connectivity + fire disturbance history	75.80	8.64	< 0.01

**Figure 3:**
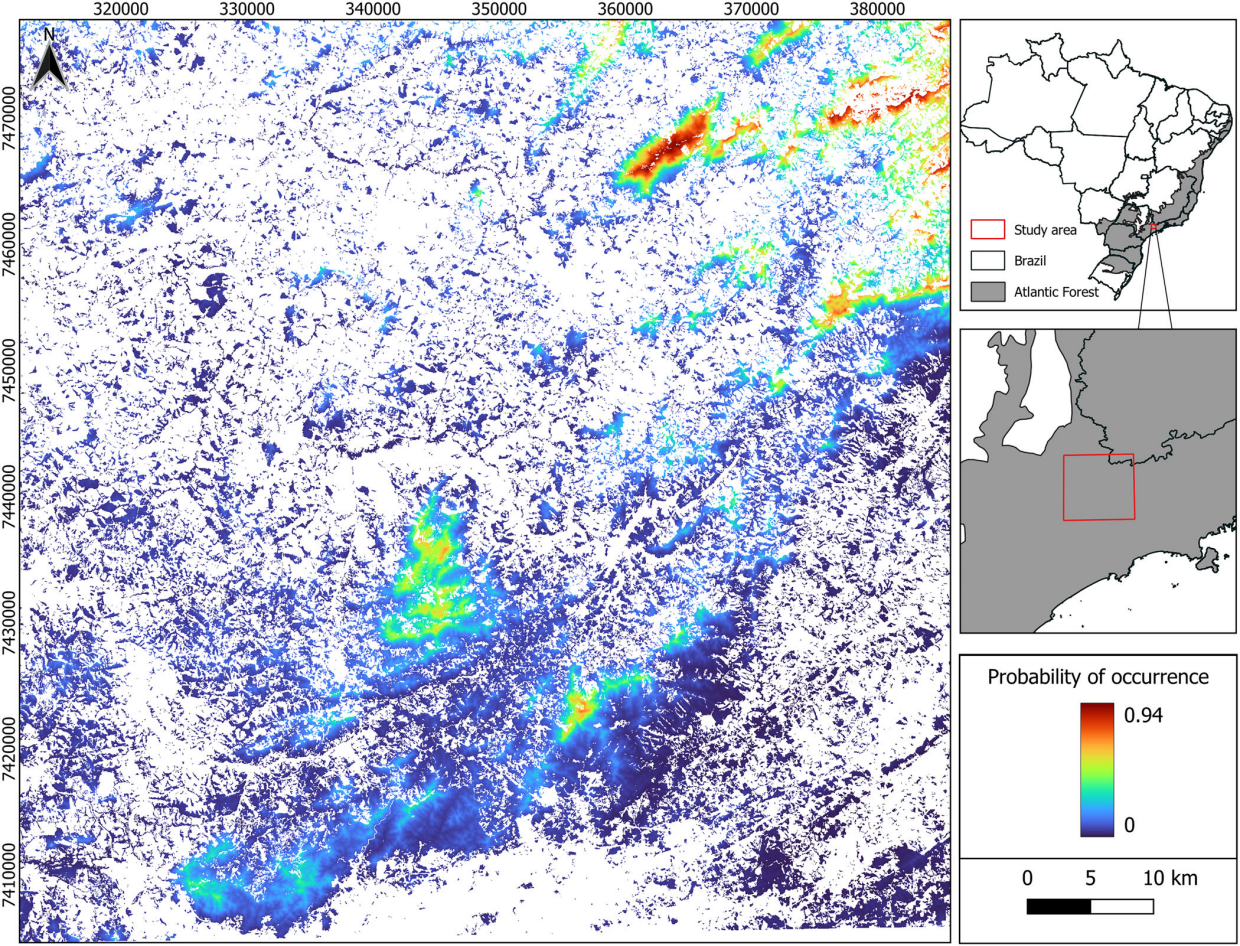
Map showing the probability of occurrence of black‐fronted titi monkeys (*Callicebus nigrifrons*) based on the best model (Model 9: forest cover 250 m + altitude), in the Cantareira‐Mantiqueira Corridor, in the states of São Paulo and Minas Gerais, Brazil, considering only forested areas. Warmer colors (closer to red) indicate higher probabilities of occurrence, whereas cooler colors (closer to blue) indicate lower probabilities.

To visualize the spatial patterns of predicted occurrence, we generated a probability surface based on this top‐ranked model (Figure 3). This map highlights areas with higher probabilities of species occurrence, particularly those characterized by high forest cover at high altitudes.

## 4 Discussion

The occurrence of black‐fronted titi monkeys (*Callicebus nigrifrons*) in the Atlantic Forest is best explained by the combined effects of forest cover and altitude. As expected, more forested areas favored the presence of the species, reinforcing the central role of habitat availability for arboreal primates. This prediction aligns with Estrada et al. (2017), who found that land‐use changes, especially livestock farming, negatively affect 59% of platyrrhine primate species primarily through forest loss and connectivity disruption. However, the positive association with altitude suggests that historical preservation at higher elevations may also play an important role.

The strong positive relationship between forest cover and black‐fronted titi monkey occurrence aligns with broader patterns observed in Callicebinae and other primates, supporting the resource availability hypothesis. Even moderate forest loss (10%) has been linked to population declines in Callicebinae (Gouveia et al. 2016), and deforestation poses a major threat to several titi monkey species, including *Plecturocebus vieirai* (Costa‐Araújo et al. 2022). However, recent studies reveal species‐specific tolerances to habitat loss. Some titi monkeys persist in small fragments if the vegetation structure remains suitable (Hilário et al. 2024; Martínez et al. 2025). For instance, *Callicebus coimbrai* relies on structural connectivity for dispersal and its densities are more influenced by understory complexity than by landscape‐scale metrics (Hilário et al. 2024), whereas *Plecturocebus olallae* depends on floristically diverse forests (Martínez et al. 2025). These patterns, together with previous studies, suggest that while habitat quantity—particularly forest cover at higher elevations—is a key driver of black‐fronted titi monkey occurrence in our study area, other factors such as vegetation structure (e.g., canopy connectivity, tree diameter) and floristic composition may still play important roles in other regions or for other Callicebinae species (e.g., Hilário et al. 2024; Martínez et al. 2025). Conservation efforts in our region should prioritize the maintenance and recovery of forest cover, particularly at higher elevations where viable populations persist. While habitat quality was not a key predictor in our models, its role in other contexts warrants further investigation (Rubio Vargas et al. 2024).

The species' preference for higher elevations likely reflects a combination of ecological and historical processes. Although mid‐ to high‐elevation zones in the Atlantic Forest are generally associated with older, structurally complex forests and greater angiosperm diversity (Amorim et al. 2009), which could support *Callicebus* diets through the production of fleshy fruits (Nagy‐Reis and Setz 2017), our findings did not reveal strong correlations between altitude and vegetation structure or quality. This suggests that other factors—such as historical patterns of land use and lower deforestation pressure in rugged terrain (Silva et al. 2007; Tabarelli et al. 2010)—may better explain the species' elevational distribution. The frugivorous–folivorous diet of *C. nigrifrons* (Nagy‐Reis and Setz 2017; Coleman and Hill 2015) may reflect ecological flexibility that enables persistence across a range of habitat conditions. Collectively, these findings highlight the conservation importance of submontane forests—particularly those between 500 and 1200 m a.s.l.—which may act as refugia in the face of ongoing environmental change (Instituto Brasileiro de Geografia e Estatística IBGE 2012).

Functional connectivity played a minimal role in predicting black‐fronted titi monkey occurrence, even when combined with forest cover. Although connectivity facilitates movement and gene flow, its influence appears weaker than that of forest cover or altitude. This may reflect the behavioral flexibility of titi monkeys, which occasionally show terrestrial behavior to access food or cross gaps (Souza‐Alves et al. 2019). Such flexibility may buffer the effects of isolation in heterogeneous landscapes. However, this potential is constrained by matrix hostility, as agricultural, pasture, and urban land uses dominate the region (MapBiomas 2023a) and are typically avoided by titi monkeys despite their occasional use of the ground (Souza‐Alves et al. 2019). Our findings align with broader evidence that configuration variables (e.g., connectivity) generally have weaker effects on primate distributions than composition variables (e.g., forest cover) (Galán‐Acedo et al. 2019c).

Vegetation quality and structure, measured by EVI and mean canopy height, also played a secondary role in predicting black‐fronted titi monkey occurrence. The relationship between vegetation quality and primate occurrence is complex, potentially due to the coarse resolution of satellite‐derived proxies, which may overlook key local attributes such as food plant richness, fruiting phenology, or microhabitat diversity. While one study report correlations between vegetation indices and primate biomass (da Silva et al. 2015), fine‐scale assessments emphasize that variables like tree size, floristic composition, and fruit availability are better predictors of primate presence (Rovero and Struhsaker 2007). The absence of strong associations between habitat quality metrics and species occurrence in our study highlights the limitations of using remotely sensed proxies alone to capture fine‐scale ecological processes. Future research combining remote sensing with field‐based vegetation assessments may provide a more comprehensive understanding of habitat suitability for *C. nigrifrons*.

The limited influence of fire disturbance history on black‐fronted titi monkey occurrence may be explained by the rarity of fire events in the study area. Over 88% of surveyed sites had no recorded fires within a 250 m radius over the past 20 years. Although fire is not part of the Atlantic Forest's natural disturbance regime (Hardesty et al. 2005), increasing drought frequency (Centro Nacional de Monitoramento e Alertas de Desastres Naturais CEMADEN 2024) may raise future fire risks. Black‐fronted titi monkeys show ecological plasticity, thriving in secondary forests (Trevelin et al. 2007; Martins 2005) and adjusting their diet during periods of scarcity (Nagy‐Reis and Setz 2017). Nevertheless, preventing fire‐related degradation remains critical for the long‐term conservation of forest‐dependent primates.

Overall, our findings demonstrate that forest cover and altitude are the primary determinants of black‐fronted titi monkey occurrence in the Atlantic Forest. Although the species shows adaptability to disturbed habitats, its long‐term persistence depends critically on forest conservation, particularly in mid‐elevation ranges (500–1200 m) that fall below the 1800 m threshold for federal permanent preservation areas under Brazil's Forest Code. These elevation zones, though not federally protected, should be prioritized through regional conservation strategies that complement national legislation. Practical measures should prioritize the protection and expansion of forest cover in mid‐ to high‐elevation areas (> 500 m), where *C. nigrifrons* occurrence is higher. These approaches align with successful primate conservation frameworks in human‐modified landscapes (Hilário et al. 2024) and could be implemented through payment for ecosystem services programs, community‐based forest management initiatives, and municipal habitat protection ordinances. Although our study assessed presence‐absence patterns rather than detailed habitat use, the results provide clear guidance for conservation planning, emphasizing the need to protect existing forest cover and restore connectivity in this vulnerable elevation range. Future research should combine these landscape‐scale findings with movement ecology data to identify optimal corridor locations and define minimum viable habitat thresholds to ensure the long‐term survival of the species in human‐modified landscapes.

## Supporting information

AJP SI MainTable.

## Data Availability

The data that support the findings of this study are openly available in Zenodo at https://doi.org/10.5281/zenodo.17136017, reference number 17136017.

## References

[ajp70079-bib-0001] Amorim, A. M. , J. G. Jardim , M. M. M. Lopes , et al. 2009. “Angiospermas Em Remanescentes De Floresta Montana No Sul Da Bahia, Brasil.” Biota Neotropica 9: 313–348. 10.1590/S1676-06032009000300028.

[ajp70079-bib-0002] Arroyo‐Rodríguez, V. , and L. Fahrig . 2014. “Why Is a Landscape Perspective Important in Studies of Primates?” American Journal of Primatology 76, no. 10: 901–909. 24715680 10.1002/ajp.22282

[ajp70079-bib-0003] Arroyo‐Rodríguez, V. , L. Fahrig , M. Tabarelli , et al. 2020. “Designing Optimal Human‐Modified Landscapes for Forest Biodiversity Conservation.” Ecology Letters 23, no. 9: 1404–1420. 10.1111/ele.13535. 32537896

[ajp70079-bib-0004] Beaudrot, L. , and A. J. Marshall . 2019. “Differences Among Regions in Environmental Predictors of Primate Community Similarity Affect Conclusions About Community Assembly.” Journal of Tropical Ecology 35, no. 2: 83–90. 10.1017/S0266467418000470.

[ajp70079-bib-0005] Benchimol, M. , and C. A. Peres . 2013. “Anthropogenic Modulators of Species–Area Relationships in Neotropical Primates: A Continental‐Scale Analysis of Fragmented Forest Landscapes.” Diversity and Distributions 19, no. 11: 1339–1352. 10.1111/ddi.12111.

[ajp70079-bib-0009] Brown, J. H. 2001. “Mammals on Mountainsides: Elevational Patterns of Diversity.” Global Ecology and Biogeography 10, no. 1: 101–109. 10.1046/j.1466-822x.2001.00228.x.

[ajp70079-bib-0010] Burnham, K. P. , and D. R. Anderson . 2002. “A Practical Information‐Theoretic Approach.” Model Selection and Multimodel Inference v. 2: 70–71. 10.1007/978-1-4757-2917-7.

[ajp70079-bib-0011] Caetano‐Andrade, V. L. , C. R. Clement , D. Weigel , et al. 2020. “Tropical Trees as Time Capsules of Anthropogenic Activity.” Trends in Plant Science 25, no. 4: 369–380. https://www.cell.com/trends/plant-science/fulltext/s1360-1385(19)30335-8. 32037081 10.1016/j.tplants.2019.12.010

[ajp70079-bib-0012] Calcagno, V. , and C. Mazancourt . 2010. “Glmulti: An R Package for Easy Automated Model Selection With (Generalized) Linear Models.” Journal of Statistical Software 34, no. 12: 1–29.

[ajp70079-bib-0013] Canale, G. R. , C. A. Peres , C. E. Guidorizzi , C. A. F. Gatto , and M. C. M. Kierulff (2012). Pervasive Defaunation of Forest Remnants in a Tropical Biodiversity Hotspot. 10.1371/journal.pone.0041671. PMC3419225 22905103

[ajp70079-bib-0014] Cardoso, F. C. G. , V. P. Zwiener , and M. C. M. Marques . 2019. “Tree Phenology Along a Successional Gradient of Tropical Atlantic Forest.” Journal of Plant Ecology 12, no. 2: 272–280. 10.1093/jpe/rty020.

[ajp70079-bib-0016] Carvalho, J. S. , B. Graham , H. Rebelo , et al. 2019. “A Global Risk Assessment of Primates under Climate and Land Use/Cover Scenarios.” Global Change Biology 25, no. 9: 3163–3178. 10.1111/gcb.14671. 31034733

[ajp70079-bib-0017] Caselli, C. B. , and E. Z. Setz . 2007. “Seasonality In Long Calls by Titi Monkeys (*Callicebus nigrifrons*) in the Atlantic Forest of Southeast Brazil.” American Journal of Primatology 69: 96–97.

[ajp70079-bib-0018] Cazetta, E. , and L. Fahrig . 2022. “The Effects of Human‐Altered Habitat Spatial Pattern on Frugivory and Seed Dispersal: A Global Meta‐Analysis.” Oikos 2022, no. 2: e08288. 10.1111/oik.08288.

[ajp70079-bib-0019] Cazzolla Gatti, R. , S. Castaldi , J. A. Lindsell , et al. 2015. “The Impact of Selective Logging and Clearcutting on Forest Structure, Tree Diversity and Above‐Ground Biomass of African Tropical Forests.” Ecological Research 30: 119–132. 10.1007/s11284-014-1217-3.

[ajp70079-bib-0020] Centro Nacional de Monitoramento e Alertas de Desastres Naturais (CEMADEN) . (2024). *Nota técnica n° 679/2024*: Análise das secas no Brasil ‐ Diagnóstico e projeções futuras. https://www.gov.br/cemaden/pt-br/assuntos/noticias-cemaden/cemaden-analisa-secas-recentes-no-brasil-e-apresenta-diagnostico-e-projecoes-como-subsidio-para-a-cop-16.

[ajp70079-bib-0021] Cochrane, M. A. 2003. “Fire Science for Rainforests.” Nature 421, no. 6926: 913–919. 10.1038/nature01437. 12606992

[ajp70079-bib-0022] Coleman, B. T. , and R. A. Hill . 2015. “Biogeographic Variation in the Diet and Behaviour of *Cercopithecus mitis* .” Folia Primatologica 85, no. 5: 319–334. 10.1159/000368895. 25591794

[ajp70079-bib-0102] Costa‐Araújo, R. , L. G. da Silva , F. R. de Melo , et al. 2022. “Primate Conservation in the Arc of Deforestation: A Case Study of Vieira's Titi Monkey *Plecturocebus vieirai* .” Oryx 56, no. 6: 837–845. 10.1017/s003060532100171x.

[ajp70079-bib-0023] Costa‐Araújo, R. , A. Luis Regolin , F. Martello , J. Pedro souza‐Alves , T. Hrbek , and M. Cezar Ribeiro . 2021. “Occurrence and Conservation of the Vulnerable Titi Monkey *Callicebus melanochir* in Fragmented Landscapes of the Atlantic Forest Hotspot.” Oryx 55, no. 6: 916–923. 10.1017/S0030605319001522.

[ajp70079-bib-0024] da Silva, L. G. , M. C. Ribeiro , É. Hasui , C. A. da Costa , and R. G. T. da Cunha . 2015. “Patch Size, Functional Isolation, Visibility and Matrix Permeability Influences Neotropical Primate Occurrence Within Highly Fragmented Landscapes.” PLoS One 10, no. 2: e0114025. 10.1371/journal.pone.0114025. 25658108 PMC4319959

[ajp70079-bib-0025] de Guinea, M. , A. Estrada , K. A. I. Nekaris , and S. Van Belle . 2019. “Arboreal Route Navigation in a Neotropical Mammal: Energetic Implications Associated With Tree Monitoring and Landscape Attributes.” Movement Ecology 7: 39. 10.1186/s40462-019-0187-z. 31890215 PMC6918719

[ajp70079-bib-0099] Estrada, A. , and P. A. Garber . 2022. “Principal Drivers and Conservation Solutions to the Impending Primate Extinction Crisis: Introduction to the Special Issue.” International Journal of Primatology 43, no. 1: 1–14. 10.1007/s10764-022-00283-1. PMC8853428 35194270

[ajp70079-bib-0027] Estrada, A. , P. A. Garber , A. B. Rylands , et al. 2017. “Impending Extinction Crisis of the World's Primates: Why Primates Matter.” Science Advances 3, no. 1: e1600946. 10.1126/sciadv.1600946. 28116351 PMC5242557

[ajp70079-bib-0028] European Space Agency . 2024. Copernicus Global Digital Elevation Model. Distributed by OpenTopography. 10.5069/G9028PQB.

[ajp70079-bib-0029] Fahrig, L. 2007. “Non‐Optimal Animal Movement in Human‐Altered Landscapes.” Functional Ecology 21, no. 6: 1003–1015. 10.1111/j.1365-2435.2007.01326.x.

[ajp70079-bib-0030] Frizzo, T. L. M. , C. Bonizário , M. P. Borges , and H. L. Vasconcelos . 2011. “Revisão Dos Efeitos Do Fogo Sobre A Fauna De Formações Savânicas Do Brasil.” Oecologia Australis 15, no. 2: 365–379. 10.4257/oeco.2011.1502.13.

[ajp70079-bib-0031] Galán‐Acedo, C. , V. Arroyo‐Rodríguez , E. Andresen , et al. 2019b. “The Conservation Value of Human‐Modified Landscapes for the World's Primates.” Nature Communications 10, no. 1: 152. 10.1038/s41467-018-08139-0. PMC6329842 30635587

[ajp70079-bib-0032] Galán‐Acedo, C. , V. Arroyo‐Rodríguez , E. Andresen , and R. Arasa‐Gisbert . 2019a. “Ecological Traits of the World's Primates.” Scientific Data 6, no. 1: 55. 10.1038/s41597-019-0059-9. 31086194 PMC6513815

[ajp70079-bib-0033] Galán‐Acedo, C. , V. Arroyo‐Rodríguez , S. J. Cudney‐Valenzuela , and L. Fahrig . 2019c. “A Global Assessment of Primate Responses to Landscape Structure.” Biological Reviews 94, no. 5: 1605–1618. 10.1111/brv.12517. 31050172

[ajp70079-bib-0034] Gestich, C. C. , V. Arroyo‐Rodríguez , M. C. Ribeiro , R. G. T. da Cunha , and E. Z. F. Setz . 2019. “Unraveling the Scales of Effect of Landscape Structure on Primate Species Richness and Density of Titi Monkeys (*Callicebus nigrifrons*).” Ecological Research 34, no. 1: 150–159. 10.1111/1440-1703.1009.

[ajp70079-bib-0035] Gestich, C. C. , C. B. Caselli , M. B. Nagy‐Reis , E. Z. F. Setz , and R. G. T. da Cunha . 2017. “Estimating Primate Population Densities: The Systematic Use of Playbacks Along Transects in Population Surveys.” American Journal of Primatology 79, no. 2: e22586. 10.1002/ajp.22586. 27464028

[ajp70079-bib-0037] Godoy, E. , B. F. C. B. Adorno , B. D. da Silva , et al. 2025. “Fire Refugia Under Threat: How Increasing Pyrodiversity Reduces Species Richness in Unburned Forests.” Forest Ecology and Management 585: 122673. 10.1016/j.foreco.2025.122673.

[ajp70079-bib-0038] Goldammer, J. G. 2016. “Fire Management in Tropical Forests.” In Tropical Forestry Handbook, 2659–2710. Springer.

[ajp70079-bib-0039] Gouveia, S. F. , J. P. Souza‐Alves , L. Rattis , et al. 2016. “Climate and Land Use Changes Will Degrade the Configuration of the Landscape for Titi Monkeys in Eastern Brazil.” Global Change Biology 22, no. 6: 2003–2012. 10.1111/gcb.13162. 26663738

[ajp70079-bib-0040] Hamard, M. , S. M. Cheyne , and V. Nijman . 2010. “Vegetation Correlates of Gibbon Density in the Peat‐Swamp Forest of the Sabangau Catchment, Central Kalimantan, Indonesia.” American Journal of Primatology 72, no. 7: 607–616. 10.1002/ajp.20815. 20186760

[ajp70079-bib-0100] Hanya, G. , and C. A. Chapman . 2012. “Linking Feeding Ecology and Population Abundance: A Review of Food Resource Limitation on Primates.” Ecological Research 28, no. 2: 183–190. Portico. 10.1007/s11284-012-1012-y.

[ajp70079-bib-0041] Hardesty, J. , R. Myers , and W. Fulks . 2005. “Fire, Ecosystems, and People: A Preliminary Assessment of Fire as a Global Conservation Issue.” George Wright Forum 22, no. 4: 78–87. http://www.jstor.org/stable/43597968.

[ajp70079-bib-0042] Hartig, F. (2022). DHARMa: *Residual Diagnostics for Hierarchical (Multi‐Level/Mixed) Regression Models* (R Package Version 0.4.6). https://CRAN.R-project.org/package=DHARMa.

[ajp70079-bib-0043] Hendershot, J. N. , J. R. Smith , C. B. Anderson , et al. 2020. “Intensive Farming Drives Long‐Term Shifts in Avian Community Composition.” Nature 579, no. 7799: 393–396. 10.1038/s41586-020-2090-6. 32188954

[ajp70079-bib-0044] Henle, K. , K. F. Davies , M. Kleyer , C. Margules , and J. Settele . 2004. “Predictors of Species Sensitivity to Fragmentation.” Biodiversity & Conservation 13: 207–251. 10.1023/B:BIOC.0000004319.91643.9e.

[ajp70079-bib-0045] Hesselbarth, M. H. K. , M. Sciaini , K. A. With , K. Wiegand , and J. Nowosad . 2019. “Landscapemetrics: An Open‐Source R Tool to Calculate Landscape Metrics.” Ecography 42, no. 10: 1648–1657. 10.1111/ecog.04617.

[ajp70079-bib-0046] Hilário, R. R. , B. Moraes , J. P. Souza‐Alves , and S. F. Ferrari . 2024. “The Density of *Callicebus coimbrai* Is Better Predicted by Vegetation Structure Variables Than by Surrounding Landscape.” International Journal of Primatology 45, no. 1: 54–71. 10.1007/s10764-022-00278-y.

[ajp70079-bib-0047] Huete, A. , K. Didan , T. Miura , E. P. Rodriguez , X. Gao , and L. G. Ferreira . 2002. “Overview of the Radiometric and Biophysical Performance of the MODIS Vegetation Indices.” Remote Sensing of Environment 83, no. 1–2: 195–213. 10.1016/S0034-4257(02)00096-2.

[ajp70079-bib-0048] Instituto Brasileiro de Geografia e Estatística (IBGE) . (2012). Manual Técnico da Vegetação Brasileira (2ª ed.). https://biblioteca.ibge.gov.br/visualizacao/livros/liv63011.pdf.

[ajp70079-bib-0049] Jackson, H. B. , and L. Fahrig . 2015. “Are Ecologists Conducting Research at the Optimal Scale?” Global Ecology and Biogeography 24, no. 1: 52–63. 10.1111/geb.12233.

[ajp70079-bib-0050] Jacques, A. V. A. 2003. “A Queima Das Pastagens Naturais: Efeitos Sobre O Solo E a Vegetação.” Ciência Rural 33: 177–181. 10.1590/S0103-84782003000100030.

[ajp70079-bib-0051] Jerusalinsky, L. , F. R. de Melo , R. A. Mittermeier , S. Quadros , and A. B. Rylands 2020. *Callicebus nigrifrons*. The IUCN Red List of Threatened Species 2020: *e*.T39943A17973667. 10.2305/IUCN.UK.2020-3.RLTS.T39943A17973667.en.

[ajp70079-bib-0052] Johnson, C. N. , A. Balmford , B. W. Brook , et al. 2017. “Biodiversity Losses and Conservation Responses in the Anthropocene.” Science 356, no. 6335: 270–275. 10.1126/science.aam9317. 28428393

[ajp70079-bib-0054] Korstjens, A. H. , J. Lehmann , and R. I. M. Dunbar . 2018. “Time Constraints Do Not Limit Group Size in Arboreal Guenons But Do Explain Community Size and Distribution Patterns.” International Journal of Primatology 39: 511–531. 10.1007/s10764-018-0048-4. PMC6182722 30369685

[ajp70079-bib-0055] Lidar, A. 2002. “Lidar Remote Sensing for Ecosystem Studies.” BioScience 52, no. 1: 19–30. https://andrewsforest.oregonstate.edu/sites/default/files/lter/pubs/pdf/pub2813.pdf.

[ajp70079-bib-0056] Lovett, G. M. , C. G. Jones , M. G. Turner , and K. C. Weathers . 2005. “Ecosystem Function in Heterogeneous Landscapes.” In Ecosystem Function in Heterogeneous Landscapes, edited by G. M. Lovett , C. G. Jones , M. G. Turner , and K. C. Weathers , 1–4. Springer. 10.1007/0-387-24091-8_1.

[ajp70079-bib-0057] Magioli, M. , K. M. P. M. Ferraz , E. Z. F. Setz , et al. 2016. “Connectivity Maintain Mammal Assemblages Functional Diversity Within Agricultural and Fragmented Landscapes.” European journal of wildlife research 62: 431–446. 10.1007/s10344-016-1017-x.

[ajp70079-bib-0058] Magioli, M. , M. Z. Moreira , R. C. B. Fonseca , M. C. Ribeiro , M. G. Rodrigues , and K. M. P. M. B. Ferraz . 2019. “Human‐Modified Landscapes Alter Mammal Resource and Habitat Use and Trophic Structure.” Proceedings of the National Academy of Sciences 116, no. 37: 18466–18472. 10.1073/pnas.1904384116. PMC6744859 31451670

[ajp70079-bib-0059] MapBiomas . (2023a). Projeto MapBiomas: Coleção BETA da Série Anual de Mapas de Cobertura e Uso de Solo do Brasil. https://mapbiomas.org.

[ajp70079-bib-0060] MapBiomas . (2023b). Projeto MapBiomas: Coleção 2.0 da Série Mapa das Cicatrizes do Fogo. https://mapbiomas.org.

[ajp70079-bib-0061] Martensen, A. C. , R. G. Pimentel , and J. P. Metzger . 2008. “Relative Effects of Fragment Size and Connectivity on Bird Community in the Atlantic Rain Forest: Implications for Conservation.” Biological Conservation 141, no. 9: 2184–2192. 10.1016/j.biocon.2008.06.008.

[ajp70079-bib-0062] Martensen, A. C. , M. C. Ribeiro , C. Banks‐Leite , P. I. Prado , and J. P. Metzger . 2012. “Associations of Forest Cover, Fragment Area, and Connectivity With Neotropical Understory Bird Species Richness and Abundance.” Conservation Biology 26, no. 6: 1100–1111. 10.1111/j.1523-1739.2012.01940.x. 23003666

[ajp70079-bib-0063] Martínez, J. , R. Márquez , A. Reinaga , M. Campera , V. Nijman , and R. B. Wallace . 2025. “Using Occupancy Modeling to Provide Insights into Suitable Habitat Characteristics for the Already Restricted and Critically Endangered Olalla's Titi Monkey (*Plecturocebus olallae*).” Primates 66, no. 1: 143–155. 10.1007/s10329-024-01171-3. 39612125 PMC11735481

[ajp70079-bib-0064] Martins, M. M. 2005. “Density of Primates in Four Semi‐Deciduous Forest Fragments of São Paulo, Brazil.” Biodiversity & Conservation 14: 2321–2329. 10.1007/s10531-004-1666-z.

[ajp70079-bib-0065] Miura, T. , A. R. Huete , W. J. D. Van Leeuwen , and K. Didan . 1998. “Vegetation Detection Through Smoke‐Filled Aviris Images: An Assessment Using Modis Band Passes.” Journal of Geophysical Research: Atmospheres 103, no. D24: 32001–32011. 10.1029/98JD00051.

[ajp70079-bib-0066] Montagnana, P. C. 2018. *Efeitos Multiescala da Quantidade de Habitat e Heterogeneidade da Paisagem Sobre Comunidade de Abelhas* (Tese de Doutorado). Universidade de São Paulo, Ribeirão Preto.

[ajp70079-bib-0067] Montes, J. 2005. “Fauna de Culicidae da Serra da Cantareira, São Paulo, Brasil.” Revista de saúde pública 39: 578–584. 10.1590/S0034-89102005000400010. 16113907

[ajp70079-bib-0068] Morellato, L. P. C. , D. C. Talora , A. Takahasi , C. C. Bencke , E. C. Romera , and V. B. Zipparro . 2000. “Phenology of Atlantic Rain Forest Trees: A Comparative Study 1.” Biotropica 32, no. 4b: 811–823. 10.1111/j.1744-7429.2000.tb00620.x.

[ajp70079-bib-0069] Müller, A. E. , and G. Anzenberger . 2002. “Duetting in the Titi Monkey *Callicebus cupreus*: Structure, Pair Specificity and Development of Duets.” Folia Primatologica 73, no. 2–3: 104–115. 10.1159/000064788. 12207057

[ajp70079-bib-0070] Myers, N. , R. A. Mittermeier , C. G. Mittermeier , G. A. B. da Fonseca , and J. Kent . 2000. “Biodiversity Hotspots for Conservation Priorities.” Nature 403: 853–858. 10.1038/35002501. 10706275

[ajp70079-bib-0071] Nagy‐Reis, M. B. , and E. Z. F. Setz . 2017. “Foraging Strategies of Black‐Fronted Titi Monkeys (*Callicebus nigrifrons*) in Relation to Food Availability in a Seasonal Tropical Forest.” Primates 58: 149–158. 10.1007/s10329-016-0556-9. 27485746

[ajp70079-bib-0072] Newbold, T. , L. N. Hudson , A. P. Arnell , et al. 2016. “Has Land Use Pushed Terrestrial Biodiversity Beyond the Planetary Boundary? A Global Assessment.” Science 353, no. 6296: 288–291. 10.1126/science.aaf2201. 27418509

[ajp70079-bib-0073] Nyirambangutse, B. , E. Zibera , F. K. Uwizeye , et al. 2017. “Carbon Stocks and Dynamics at Different Successional Stages in an Afromontane Tropical Forest.” Biogeosciences 14, no. 5: 1285–1303. 10.5194/bg-14-1285-2017.

[ajp70079-bib-0074] Ogaya, R. , A. Barbeta , C. Başnou , and J. Peñuelas . 2015. “Satellite Data as Indicators of Tree Biomass Growth and Forest Dieback in a Mediterranean Holm Oak Forest.” Annals of Forest Science 72: 135–144. 10.1007/s13595-014-0408-y.

[ajp70079-bib-0075] Pardini, R. , D. Faria , G. M. Accacio , et al. 2009. “The Challenge of Maintaining Atlantic Forest Biodiversity: A Multi‐Taxa Conservation Assessment of Specialist and Generalist Species in an Agro‐Forestry Mosaic in Southern Bahia.” Biological Conservation 142, no. 6: 1178–1190. 10.1016/j.biocon.2009.02.010.

[ajp70079-bib-0077] Pebsworth, P. A. , M. A. Huffman , J. E. Lambert , and S. L. Young . 2019. “Geophagy Among Nonhuman Primates: A Systematic Review of Current Knowledge and Suggestions for Future Directions.” American Journal of Physical Anthropology 168: 164–194. 10.1002/ajpa.23724. 30508222

[ajp70079-bib-0078] Pereira, L. A. , V. E. W. Campos , C. C. Gestich , M. C. Ribeiro , and L. Culot . 2022. “Erosion of Primate Functional Diversity in Small and Isolated Forest Patches Within Movement‐Resistant Landscapes.” Animal Conservation 25, no. 6: 782–795. 10.1111/acv.12784.

[ajp70079-bib-0079] Polis, G. A. , Power, M. E. , and Huxel, G. R. , ed. 2004. Food Webs at the Landscape Level. University of Chicago Press.

[ajp70079-bib-0080] R Core Team . 2024. R: A Language and Environment for Statistical Computing (Version 4.4.1). R Foundation for Statistical Computing. https://www.R-project.org.

[ajp70079-bib-0081] Rezende, V. L. , P. V. Eisenlohr , A. C. Vibrans , and A. T. de Oliveira‐Filho . 2015. “Humidity, Low Temperature Extremes, and Space Influence Floristic Variation Across an Insightful Gradient in the Subtropical Atlantic Forest.” Plant Ecology 216: 759–774. 10.1007/s11258-015-0465-9.

[ajp70079-bib-0082] Roberts, P. , R. Hamilton , and D. R. Piperno . 2021. “Tropical Forests as Key Sites of the “Anthropocene”: Past and Present Perspectives.” Proceedings of the National Academy of Sciences 118, no. 40: e2109243118. 10.1073/pnas.2109243118. PMC8501787 34580229

[ajp70079-bib-0083] Rodrigues, R. R. (2008). Diretrizes Para a Conservação e Restauração da Biodiversidade no Estado de São Paulo. https://repositorio.cetesb.sp.gov.br/handle/123456789/3955.

[ajp70079-bib-0084] Rovero, F. , and T. T. Struhsaker . 2007. “Vegetative Predictors of Primate Abundance: Utility and Limitations of a Fine‐Scale Analysis.” American Journal of Primatology 69, no. 11: 1242–1256. 10.1002/ajp.20431. 17358022

[ajp70079-bib-0086] Rubio Vargas, C. L. , Z. L. Cerón Cancharis , and E. W. Heymann . 2024. “Characterization of Forest Fragments Occupied by the Critically Endangered and Endemic San Martín Titi Monkey (*Plecturocebus oenanthe)* .” Frontiers in Conservation Science 5: 1401517. 10.3389/fcosc.2024.1401517.

[ajp70079-bib-0088] Silva, W. , J. Metzger , S. Simões , and C. Simonetti . 2007. “Relief Influence on the Spatial Distribution of the Atlantic Forest Cover on the Ibiúna Plateau, SP.” Brazilian Journal of Biology 67: 403–411. 10.1590/S1519-69842007000300004. 18094822

[ajp70079-bib-0090] Souza‐Alves, J. P. , I. Mourthe , R. R. Hilário , et al. 2019. “Terrestrial Behavior In Titi Monkeys (*Callicebus*, *Cheracebus*, and *Plecturocebus*): Potential Correlates, Patterns, and Differences Between Genera.” International Journal of Primatology 40: 553–572. 10.1007/s10764-019-00105-x.

[ajp70079-bib-0091] Tabarelli, M. , A. V. Aguiar , M. C. Ribeiro , J. P. Metzger , and C. A. Peres . 2010. “Prospects for Biodiversity Conservation in the Atlantic Forest: Lessons From Aging Human‐Modified Landscapes.” Biological Conservation 143, no. 10: 2328–2340.

[ajp70079-bib-0092] Tolan, J. , H. I. Yang , B. Nosarzewski , et al. 2024. “Very High Resolution Canopy Height Maps From Rgb Imagery Using Self‐Supervised Vision Transformer and Convolutional Decoder Trained on Aerial Lidar.” Remote Sensing of Environment 300: 113888. 10.1016/j.rse.2023.113888.

[ajp70079-bib-0093] Trevelin, L. C. , M. Port‐Carvalho , M. Silveira , and E. Morell . 2007. “Abundance, Habitat Use and Diet of *Callicebus nigrifrons* Spix (Primates, Pitheciidae) in Cantareira State Park, São Paulo, Brazil.” Revista Brasileira de Zoologia 24, no. 4: 1071–1077. 10.1590/S0101-81752007000400026.

[ajp70079-bib-0094] Urban, D. , and T. Keitt . 2001. “Landscape Connectivity: A Graph‐Theoretic Perspective.” Ecology 82, no. 5: 1205–1218. 10.1890/0012-9658(2001)082[1205:LCAGTP]2.0.CO;2.

[ajp70079-bib-0095] Wang, J. , B. Gao , and A. Stein . 2020. “The Spatial Statistic Trinity: A Generic Framework for Spatial Sampling and Inference.” Environmental Modelling & Software 134: 104835. 10.1016/j.envsoft.2020.104835.

[ajp70079-bib-0096] Watling, J. I. , A. J. Nowakowski , M. A. Donnelly , and J. L. Orrock . 2011. “Meta‐Analysis Reveals the Importance of Matrix Composition for Animals in Fragmented Habitat.” Global Ecology and Biogeography 20, no. 2: 209–217. 10.1111/j.1466-8238.2010.00586.x.

[ajp70079-bib-0098] Wieczkowski, J. 2004. “Ecological Correlates of Abundance in the Tana Mangabey (*Cercocebus galeritus*).” American Journal of Primatology 63, no. 3: 125–138. 10.1002/ajp.20046. 15258957

