# Supplementary material for: Forest Cover and Altitude Are Key to the Occurrence of Black‐Fronted Titi Monkeys (Callicebus nigrifrons) in the Brazilian Atlantic Forest
Source: Am J Primatol. 2025 Sep 19;87(9):e70079. doi: 10.1002/ajp.70079 (PMC12447785; doi:10.1002/ajp.70079)
Supplement: Supplementary file 1 — AJP SI MainTable. [file AJP-87-e70079-s001.docx]

**Table S1** – Data collection in the field

| **Long** | **Lat** | **Date** | **Hora** | **Occ** | **Date** | **Hora** | **Occ** | **Date** | **Hora** | **Occ** |
| --- | --- | --- | --- | --- | --- | --- | --- | --- | --- | --- |
| -46.61313 | -23.42667 | 08/03/2022 | 11:03 | 0 | 22/03/2022 | 17:29 | 1 | NA | NA | NA |
| -46.67966 | -23.41693 | 08/03/2022 | 07:57 | 1 | NA | NA | NA | NA | NA | NA |
| -46.56459 | -23.37013 | 09/03/2022 | 07:51 | 0 | 23/03/2022 | 08:08 | 0 | 13/11/2023 | 06:48 | 0 |
| -46.51980 | -23.34770 | 09/03/2022 | 09:29 | 0 | 23/03/2022 | 10:45 | 1 | NA | NA | NA |
| -46.56088 | -23.33864 | 09/03/2022 | 11:19 | 0 | 23/03/2022 | 07:44 | 1 | NA | NA | NA |
| -46.62886 | -23.39491 | 09/03/2022 | 06:51 | 1 | NA | NA | NA | NA | NA | NA |
| -46.68386 | -23.00138 | 10/03/2022 | 08:00 | 0 | 06/06/2022 | 12:20 | 0 | 23/10/2023 | 08:03 | 0 |
| -46.64865 | -22.97482 | 10/03/2022 | 09:28 | 0 | 14/07/2023 | 08:43 | 0 | 23/10/2023 | 09:09 | 0 |
| -46.67059 | -23.01033 | 10/03/2022 | 06:55 | 1 | NA | NA | NA | NA | NA | NA |
| -46.63002 | -22.99701 | 10/03/2022 | 11:09 | 1 | NA | NA | NA | NA | NA | NA |
| -46.25460 | -22.81873 | 11/03/2022 | 10:17 | 1 | NA | NA | NA | NA | NA | NA |
| -46.30730 | -22.87542 | 11/03/2022 | 06:29 | 1 | NA | NA | NA | NA | NA | NA |
| -46.29677 | -22.85906 | 11/03/2022 | 08:34 | 1 | NA | NA | NA | NA | NA | NA |
| -46.52490 | -23.37940 | 23/03/2022 | 11:33 | 1 | NA | NA | NA | NA | NA | NA |
| -46.66310 | -23.36740 | 24/03/2022 | 10:42 | 1 | NA | NA | NA | NA | NA | NA |
| -46.49826 | -23.35358 | 04/04/2022 | 08:38 | 1 | NA | NA | NA | NA | NA | NA |
| -46.24233 | -22.88441 | 20/04/2022 | 11:21 | 1 | NA | NA | NA | NA | NA | NA |
| -46.14750 | -23.02580 | 17/05/2022 | 11:33 | 1 | NA | NA | NA | NA | NA | NA |
| -46.28520 | -23.00250 | 19/05/2022 | NA | 0 | 12/07/2023 | 10:58 | 0 | 25/10/2023 | 09:14 | 0 |
| -46.27920 | -22.95570 | 19/05/2022 | 15:04 | 1 | NA | NA | NA | NA | NA | NA |
| -46.30470 | -23.01390 | 19/05/2022 | NA | 1 | NA | NA | NA | NA | NA | NA |
| -46.74210 | -23.02726 | 06/06/2022 | 11:15 | 0 | 20/10/2022 | 06:15 | 0 | 16/01/2023 | 06:15 | 0 |
| -46.82399 | -23.04734 | 06/06/2022 | 09:04 | 0 | 25/08/2022 | 11:54 | 0 | 20/10/2022 | 08:27 | 0 |
| -46.65946 | -23.28665 | 07/06/2022 | 12:45 | 1 | NA | NA | NA | NA | NA | NA |
| -46.26542 | -23.31395 | 08/06/2022 | 08:23 | 0 | 16/10/2022 | 11:04 | 0 | 26/10/2023 | 06:47 | 0 |
| -46.29003 | -23.26607 | 08/06/2022 | 11:16 | 0 | 13/07/2023 | 09:06 | 0 | 13/11/2023 | 10:20 | 1 |
| -46.12231 | -23.30824 | 09/06/2022 | 07:52 | 0 | 26/08/2022 | 07:37 | 0 | 16/10/2022 | 07:36 | 0 |
| -46.15537 | -23.33288 | 09/06/2022 | 10:33 | 0 | 26/08/2022 | 10:28 | 0 | 16/10/2022 | 09:00 | 0 |
| -46.36890 | -22.89590 | 27/06/2022 | NA | 0 | 13/07/2023 | 10:55 | 1 | NA | NA | NA |
| -46.60600 | -23.26250 | 28/06/2022 | NA | 0 | 15/01/2023 | 06:18 | 0 | 24/10/2023 | 06:25 | 0 |
| -46.32190 | -23.13900 | 30/06/2022 | NA | 0 | 12/07/2023 | 07:55 | 0 | 12/11/2023 | 07:44 | 0 |
| -46.29780 | -23.13140 | 30/06/2022 | NA | 0 | 16/01/2023 | 11:00 | 0 | 12/11/2023 | 08:10 | 0 |
| -46.49550 | -23.16610 | 30/06/2022 | 10:59 | 1 | NA | NA | NA | NA | NA | NA |
| -46.62090 | -23.40750 | 06/07/2022 | 12:24 | 1 | NA | NA | NA | NA | NA | NA |
| -46.79038 | -23.02844 | 25/08/2022 | 09:35 | 0 | 16/01/2023 | 08:43 | 0 | 23/10/2023 | 06:45 | 0 |
| -46.15767 | -23.30648 | 26/08/2022 | 09:08 | 0 | 13/07/2023 | 10:14 | 0 | 26/10/2023 | 11:00 | 0 |
| -46.15314 | -23.27799 | 26/08/2022 | 12:00 | 0 | 13/07/2023 | 11:18 | 0 | 26/10/2023 | 08:20 | 0 |
| -46.43975 | -23.03420 | 27/08/2022 | 07:45 | 1 | NA | NA | NA | NA | NA | NA |
| -46.42284 | -23.04821 | 27/08/2022 | 09:11 | 1 | NA | NA | NA | NA | NA | NA |
| -46.72984 | -23.13445 | 28/08/2022 | 07:55 | 0 | 16/01/2023 | 10:35 | 0 | 24/10/2023 | 10:57 | 0 |
| -46.68066 | -23.12662 | 28/08/2022 | 10:26 | 0 | 24/01/2023 | 09:45 | 0 | 20/02/2023 | 10:40 | 0 |
| -46.75270 | -23.15735 | 28/08/2022 | 08:51 | 0 | 24/01/2023 | 10:50 | 0 | 20/02/2023 | 09:30 | 1 |
| -46.72308 | -23.16855 | 28/08/2022 | 07:14 | 0 | 16/01/2023 | 10:04 | 0 | 24/10/2023 | 08:18 | 1 |
| -46.13973 | -22.91906 | 30/08/2022 | 09:47 | 0 | 14/10/2022 | 10:48 | 0 | 25/10/2023 | 11:00 | 0 |
| -46.31060 | -23.16267 | 02/09/2022 | 10:00 | 1 | NA | NA | NA | NA | NA | NA |
| -46.51518 | -23.19434 | 27/09/2022 | 07:45 | 1 | NA | NA | NA | NA | NA | NA |
| -46.2828 | -23.28661 | 08/10/2022 | 06:34 | 1 | NA | NA | NA | NA | NA | NA |
| -46.41376 | -23.3338 | 13/10/2022 | 10:14 | 1 | NA | NA | NA | NA | NA | NA |
| -46.36912 | -23.28324 | 13/10/2022 | 11:30 | 1 | NA | NA | NA | NA | NA | NA |
| -46.12950 | -22.95900 | 14/10/2022 | 09:07 | 1 | NA | NA | NA | NA | NA | NA |
| -46.35156 | -23.15874 | 15/10/2022 | 07:47 | 1 | NA | NA | NA | NA | NA | NA |
| -46.19936 | -23.07328 | 15/10/2022 | 10:06 | 1 | NA | NA | NA | NA | NA | NA |
| -46.39265 | -22.88424 | 17/10/2022 | 07:15 | 0 | 13/12/2022 | 15:33 | 1 | NA | NA | NA |
| -46.36325 | -22.94429 | 17/10/2022 | 10:06 | 1 | NA | NA | NA | NA | NA | NA |
| -46.46401 | -23.05025 | 19/10/2022 | 11:35 | 1 | NA | NA | NA | NA | NA | NA |
| -46.40511 | -23.02643 | 19/10/2022 | 08:34 | 1 | NA | NA | NA | NA | NA | NA |
| -46.33900 | -22.89870 | 15/11/2022 | 13:15 | 1 | NA | NA | NA | NA | NA | NA |
| -46.35161 | -23.17663 | 26/11/2022 | 08:30 | 0 | 14/12/2022 | 11:45 | 1 | NA | NA | NA |
| -46.31707 | -23.02134 | 27/11/2022 | 08:30 | 0 | 12/07/2023 | 09:55 | 0 | 25/10/2023 | 07:30 | 0 |
| -46.43433 | -23.00392 | 27/11/2022 | 11:30 | 0 | 13/07/2023 | 09:54 | 0 | 23/10/2023 | 10:27 | 0 |
| -46.40943 | -23.29119 | 28/11/2022 | 08:30 | 0 | 13/07/2023 | 07:40 | 0 | 13/11/2023 | 08:30 | 1 |
| -46.74121 | -23.12424 | 13/12/2022 | 10:30 | 0 | 13/07/2023 | 07:40 | 0 | 24/10/2023 | 09:52 | 0 |
| -46.29413 | -22.97413 | 15/12/2022 | 07:30 | 1 | NA | NA | NA | NA | NA | NA |
| -46.55673 | -23.18541 | 14/01/2023 | 10:36 | 0 | 20/02/2023 | 07:30 | 0 | 27/05/2023 | 14:05 | 1 |
| -46.52495 | -23.18674 | 15/01/2023 | 10:20 | 1 | NA | NA | NA | NA | NA | NA |
| -46.62657 | -23.25905 | 15/01/2023 | 07:08 | 1 | NA | NA | NA | NA | NA | NA |
| -46.63859 | -23.25331 | 15/01/2023 | 08:21 | 1 | NA | NA | NA | NA | NA | NA |
| -46.16455 | -23.05643 | 16/01/2023 | 07:20 | 0 | 14/07/2023 | 07:40 | 1 | NA | NA | NA |
| -46.15634 | -23.07763 | 16/01/2023 | 09:00 | 0 | 14/07/2023 | 08:12 | 0 | 12/11/2023 | 10:20 | 0 |
| -46.78284 | -23.06423 | 16/01/2023 | 07:33 | 1 | NA | NA | NA | NA | NA | NA |
| -46.36367 | -22.92057 | 17/01/2023 | NA | 1 | NA | NA | NA | NA | NA | NA |
| -46.35773 | -22.90157 | 18/01/2023 | 17:30 | 1 | NA | NA | NA | NA | NA | NA |

† Long: longitude, Lat: latitude, Occ: occurrence.

**Table S2** – Variable values

| **Long** | **Lat** | **Site** | **FC_250m** | **FC_500m** | **FC_750m** | **FC_1000m** | **Con_100m** | **EVI** | **Alt_30m** | **MCH_1m** | **BA_250m** |
| --- | --- | --- | --- | --- | --- | --- | --- | --- | --- | --- | --- |
| -46.74121 | -23.12424 | 11 | 59.91 | 31.81 | 22.20 | 18.41 | 159773 | 0.50 | 868.80 | 7.83 | 0.00 |
| -46.72984 | -23.13445 | 13 | 46.20 | 36.01 | 30.73 | 24.46 | 159773 | 0.47 | 826.33 | 6.09 | 0.43 |
| -46.68066 | -23.12662 | 15 | 85.53 | 51.77 | 44.99 | 38.07 | 1337 | 0.56 | 847.22 | 12.38 | 0.00 |
| -46.75270 | -23.15735 | 16 | 81.57 | 78.21 | 70.63 | 58.85 | 159773 | 0.53 | 845.55 | 12.39 | 0.00 |
| -46.72308 | -23.16855 | 19 | 75.77 | 47.37 | 39.87 | 37.90 | 159773 | 0.52 | 843.69 | 10.23 | 0.00 |
| -46.67059 | -23.01033 | 22 | 33.92 | 24.69 | 15.94 | 11.95 | 28 | 0.44 | 780.81 | 5.30 | 0.00 |
| -46.63002 | -22.99701 | 23 | 79.55 | 29.65 | 15.92 | 13.24 | 368 | 0.53 | 813.99 | 7.81 | 0.00 |
| -46.68386 | -23.00138 | 25 | 91.28 | 77.97 | 78.66 | 71.20 | 726 | 0.50 | 816.13 | 11.99 | 0.00 |
| -46.64865 | -22.97482 | 30 | 32.29 | 14.27 | 15.33 | 12.00 | 8 | 0.48 | 820.53 | 3.85 | 0.00 |
| -46.33900 | -22.89870 | 31 | 84.27 | 90.99 | 94.28 | 93.99 | 159773 | 0.50 | 1584.25 | 8.45 | 0.00 |
| -46.36890 | -22.89590 | 32 | 28.62 | 17.13 | 17.99 | 23.49 | 159773 | 0.42 | 949.63 | 3.79 | 0.00 |
| -46.39265 | -22.88424 | 35 | 40.77 | 12.57 | 9.48 | 11.00 | 104 | 0.43 | 856.14 | 3.89 | 0.09 |
| -46.35773 | -22.90157 | 36 | 88.25 | 94.59 | 92.93 | 86.13 | 159773 | 0.53 | 1260.68 | 6.84 | 0.00 |
| -46.36325 | -22.94429 | 37 | 53.21 | 34.51 | 26.68 | 24.42 | 159773 | 0.54 | 947.56 | 6.04 | 0.00 |
| -46.36367 | -22.92057 | 38 | 92.08 | 80.96 | 78.82 | 76.59 | 159773 | 0.59 | 1042.80 | 10.88 | 0.00 |
| -46.78284 | -23.06423 | 42 | 59.57 | 39.99 | 35.37 | 25.97 | 88 | 0.50 | 870.07 | 8.35 | 0.00 |
| -46.74210 | -23.02726 | 43 | 67.78 | 35.72 | 24.24 | 22.47 | 53 | 0.48 | 785.63 | 9.10 | 0.00 |
| -46.82399 | -23.04734 | 45 | 44.89 | 14.29 | 6.43 | 3.73 | 13 | 0.54 | 814.86 | 6.39 | 0.00 |
| -46.79038 | -23.02844 | 47 | 7.69 | 4.37 | 4.71 | 4.92 | 5 | 0.43 | 834.28 | 2.23 | 0.00 |
| -46.52490 | -23.37940 | 52 | 100.00 | 100.00 | 100.00 | 100.00 | 159773 | 0.53 | 853.25 | 15.14 | 0.00 |
| -46.56459 | -23.37013 | 53 | 97.58 | 87.14 | 81.00 | 77.28 | 159773 | 0.54 | 1084.59 | 14.20 | 0.00 |
| -46.51980 | -23.34770 | 56 | 100.00 | 98.49 | 89.58 | 84.73 | 159773 | 0.55 | 1059.10 | 10.69 | 0.00 |
| -46.56088 | -23.33864 | 59 | 96.22 | 74.04 | 63.11 | 60.92 | 159773 | 0.59 | 884.50 | 14.21 | 0.00 |
| -46.49826 | -23.35358 | 60 | 96.32 | 95.77 | 93.09 | 90.54 | 159773 | 0.49 | 1125.64 | 11.73 | 0.00 |
| -46.41376 | -23.3338 | 64 | 97.76 | 95.74 | 95.15 | 94.85 | 159773 | 0.49 | 854.82 | 10.67 | 0.00 |
| -46.36912 | -23.28324 | 65 | 92.79 | 87.17 | 92.58 | 95.32 | 159773 | 0.58 | 959.52 | 13.41 | 0.00 |
| -46.40943 | -23.29119 | 66 | 96.05 | 88.04 | 86.65 | 86.36 | 159773 | 0.56 | 1263.92 | 10.89 | 0.00 |
| -46.31707 | -23.02134 | 73 | 56.75 | 50.38 | 38.79 | 38.40 | 159773 | 0.64 | 1083.27 | 10.04 | 0.63 |
| -46.29413 | -22.97413 | 74 | 96.35 | 89.54 | 67.84 | 52.69 | 159773 | 0.59 | 1044.88 | 12.62 | 0.00 |
| -46.28520 | -23.00250 | 76 | 38.66 | 22.04 | 21.96 | 26.65 | 159773 | 0.54 | 944.07 | 5.89 | 0.00 |
| -46.27920 | -22.95570 | 79 | 46.42 | 20.20 | 15.86 | 14.09 | 34 | 0.53 | 1024.48 | 6.71 | 0.00 |
| -46.30470 | -23.01390 | 80 | 82.86 | 70.54 | 58.62 | 44.58 | 159773 | 0.57 | 1069.26 | 10.72 | 0.00 |
| -46.52495 | -23.18674 | 81 | 87.57 | 93.72 | 96.65 | 97.33 | 159773 | 0.53 | 1238.78 | 9.28 | 0.00 |
| -46.51518 | -23.19434 | 83 | 99.96 | 97.98 | 97.80 | 97.70 | 159773 | 0.53 | 1142.46 | 10.70 | 0.00 |
| -46.49550 | -23.16610 | 87 | 100.00 | 95.77 | 87.77 | 81.05 | 159773 | 0.56 | 940.58 | 10.54 | 0.00 |
| -46.55673 | -23.18541 | 88 | 45.28 | 29.91 | 24.12 | 24.10 | 268 | 0.53 | 841.87 | 4.28 | 0.00 |
| -46.43975 | -23.03420 | 93 | 84.13 | 44.95 | 42.73 | 35.24 | 1308 | 0.58 | 989.23 | 9.87 | 0.00 |
| -46.42284 | -23.04821 | 94 | 83.16 | 72.47 | 71.41 | 64.77 | 1308 | 0.54 | 932.62 | 6.02 | 0.00 |
| -46.46401 | -23.05025 | 96 | 53.71 | 23.75 | 14.85 | 13.52 | 106 | 0.51 | 870.33 | 6.66 | 0.31 |
| -46.40511 | -23.02643 | 99 | 95.39 | 84.27 | 74.28 | 60.92 | 1308 | 0.58 | 981.24 | 8.61 | 0.00 |
| -46.42751 | -23.00672 | 100 | 21.30 | 17.63 | 16.04 | 18.76 | 137 | 0.33 | 855.55 | 1.94 | 0.00 |
| -46.65946 | -23.28665 | 112 | 91.38 | 76.05 | 64.14 | 59.50 | 159773 | 0.51 | 886.97 | 13.82 | 0.00 |
| -46.62657 | -23.25905 | 116 | 81.02 | 57.11 | 55.81 | 51.99 | 159773 | 0.53 | 927.42 | 9.70 | 0.00 |
| -46.60600 | -23.26250 | 117 | 58.55 | 36.06 | 32.64 | 31.40 | 159773 | 0.49 | 952.24 | 7.34 | 0.00 |
| -46.63859 | -23.25331 | 119 | 97.59 | 78.76 | 66.35 | 62.95 | 159773 | 0.54 | 965.83 | 14.15 | 0.00 |
| -46.26542 | -23.31395 | 126 | 98.51 | 68.91 | 51.28 | 53.84 | 159773 | 0.54 | 670.90 | 10.84 | 0.00 |
| -46.2828 | -23.28661 | 128 | 100.00 | 87.41 | 72.65 | 61.73 | 159773 | 0.53 | 676.75 | 10.77 | 0.00 |
| -46.29003 | -23.26607 | 130 | 90.60 | 77.11 | 67.58 | 61.11 | 159773 | 0.50 | 709.46 | 9.37 | 0.00 |
| -46.12950 | -22.95900 | 137 | 80.19 | 58.20 | 42.45 | 39.55 | 159773 | 0.53 | 1391.57 | 7.92 | 1.09 |
| -46.13973 | -22.91906 | 138 | 13.08 | 12.78 | 24.09 | 22.82 | 4 | 0.42 | 1216.71 | 1.81 | 0.00 |
| -46.14750 | -23.02580 | 142 | 100.00 | 95.81 | 92.41 | 93.12 | 159773 | 0.48 | 940.23 | 9.70 | 0.00 |
| -46.16455 | -23.05643 | 147 | 76.91 | 64.70 | 65.41 | 63.81 | 159773 | 0.54 | 985.04 | 9.83 | 0.00 |
| -46.31060 | -23.16267 | 149 | 89.00 | 72.95 | 63.41 | 61.78 | 159773 | 0.58 | 940.47 | 10.82 | 0.00 |
| -46.35161 | -23.17663 | 151 | 94.86 | 87.92 | 74.66 | 66.61 | 159773 | 0.53 | 837.11 | 9.80 | 0.00 |
| -46.35156 | -23.15874 | 152 | 69.26 | 42.11 | 31.94 | 31.29 | 159773 | 0.49 | 814.51 | 7.58 | 0.00 |
| -46.32190 | -23.13900 | 155 | 51.62 | 52.96 | 53.02 | 58.98 | 159773 | 0.29 | 797.51 | 4.92 | 0.00 |
| -46.29780 | -23.13140 | 157 | 58.35 | 45.91 | 38.46 | 34.69 | 159773 | 0.29 | 816.37 | 6.67 | 0.00 |
| -46.15767 | -23.30648 | 159 | 43.02 | 16.50 | 13.44 | 7.67 | 23 | 0.52 | 703.05 | 5.89 | 0.00 |
| -46.12231 | -23.30824 | 160 | 72.11 | 61.47 | 59.49 | 46.12 | 453 | 0.48 | 687.31 | 9.04 | 0.00 |
| -46.15537 | -23.33288 | 163 | 67.18 | 45.80 | 39.76 | 32.87 | 453 | 0.54 | 721.16 | 8.19 | 0.00 |
| -46.15314 | -23.27799 | 164 | 39.11 | 20.53 | 10.22 | 7.88 | 18 | 0.47 | 692.23 | 4.64 | 0.00 |
| -46.24233 | -22.88441 | 168 | 73.62 | 49.88 | 27.52 | 23.27 | 47 | 0.59 | 1097.65 | 3.70 | 0.00 |
| -46.25460 | -22.81873 | 169 | 88.28 | 77.09 | 71.14 | 70.07 | 1872 | 0.49 | 1349.89 | 7.84 | 1.75 |
| -46.30730 | -22.87542 | 173 | 100.00 | 100.00 | 98.15 | 93.44 | 159773 | 0.52 | 1480.36 | 10.89 | 0.00 |
| -46.29677 | -22.85906 | 177 | 68.94 | 58.75 | 57.96 | 49.55 | 159773 | 0.53 | 1203.76 | 4.38 | 1.02 |
| -46.62886 | -23.39491 | 189 | 81.95 | 79.31 | 69.66 | 68.00 | 159773 | 0.50 | 991.14 | 10.13 | 0.00 |
| -46.66310 | -23.36740 | 190 | 58.58 | 68.04 | 48.21 | 45.10 | 159773 | 0.52 | 817.62 | 5.92 | 1.22 |
| -46.62090 | -23.40750 | 191 | 95.48 | 86.45 | 82.44 | 85.63 | 159773 | 0.46 | 1109.38 | 15.12 | 0.00 |
| -46.67966 | -23.41693 | 194 | 97.58 | 90.95 | 86.89 | 85.88 | 159773 | 0.52 | 1073.15 | 15.40 | 0.00 |
| -46.61313 | -23.42667 | 195 | 99.74 | 99.62 | 99.23 | 98.80 | 159773 | 0.51 | 973.00 | 14.07 | 0.00 |
| -46.19936 | -23.07328 | 199 | 80.83 | 71.37 | 78.37 | 70.53 | 159773 | 0.51 | 904.70 | 10.66 | 0.00 |
| -46.15634 | -23.07763 | 200 | 37.36 | 30.41 | 37.42 | 38.74 | 159773 | 0.46 | 719.70 | 3.32 | 0.00 |
| Average | | | 73.27 | 60.10 | 54.60 | 51.35 | 106655.18 | 0.51 | 951.47 | 8.79 | 0.10 |
| Standard deviation | | | 24.60 | 28.76 | 29.13 | 28.85 | 75647.79 | 0.06 | 189.26 | 3.42 | 0.37 |

† Long: longitude, Lat: latitude, FC: forest cover, Con: functional connectivity, Alt: altitude, MCH: mean canopy height, BA: burned area.

**Table S3** – R² values and significance levels for predictors

| **Scale** | **R²** | **p** |
| --- | --- | --- |
| Forest cover – 250m | 0.28 | < 0.00001 |
| Forest cover – 500m | 0.26 | < 0.00001 |
| Forest cover – 750m | 0.22 | < 0.0001 |
| Forest cover – 1000m | 0.21 | < 0.0001 |
| Functional connectivity – 100m | 0.12 | < 0.01 |
| EVI | 0.14 | < 0.01 |
| Altitude | 0.19 | < 0.01 |
| Mean canopy height | 0.13 | < 0.01 |
| Fire disturbance history | 0.009 | 0.34 |

**Table S4 –** Variance Inflation Factor (VIF) values

| **Variables** | **VIF** |
| --- | --- |
| Forest cover (250m) | 1.72 |
| Functional connectivity (100m) | 1.30 |
| Fire disturbance history | 1.08 |
| Altitude | 1.10 |
| EVI | 1.46 |

**Table S5** – Pearson correlation between the tested variables

| **Variables** | **r** | **t** | **df** | **p** | **95% CI** |
| --- | --- | --- | --- | --- | --- |
| Forest cover (250m) + Functional connectivity | 0.60 | 6.24 | 70 | < 0.001 | [0.32, 0.66] |
| Forest cover (250m) + EVI | 0.51 | 4.99 | 70 | < 0.001 | [0.42, 0.73] |
| Forest cover (250m) + Altitude | 0.29 | 2.53 | 70 | 0.01 | [0.06, 0.49] |
| Forest cover (250m) + Fire disturbance history | -0.03 | -0.30 | 70 | 0.75 | [-0.26, 0.19] |
| Forest cover (250m) + Mean canopy height | 0.84 | 13.24 | 70 | < 0.001 | [0.76, 0.90] |
| Functional connectivity + EVI | 0.19 | 1.59 | 70 | 0.11 | [-0.04, 0.40] |
| Functional connectivity + Altitude | 0.26 | 2.30 | 70 | 0.02 | [0.03, 0.46] |
| Functional connectivity + Fire disturbance history | 0.08 | 0.65 | 70 | 0.51 | [-0.15, 0.30] |
| Functional connectivity + Mean canopy height | 0.55 | 5.48 | 70 | < 0.001 | [0.36, 0.69] |
| Altitude + EVI | 0.22 | 1.89 | 70 | 0.06 | [-0.01, 0.43] |
| Altitude + Fire disturbance history | 0.30 | 2.69 | 70 | < 0.01 | [0.08, 0.50] |
| Altitude + Mean canopy height | 0.13 | 1.16 | 70 | 0.25 | [-0.09, 0.35] |
| Mean canopy height + EVI | 0.46 | 4.40 | 70 | < 0.001 | [0.26, 0.63] |
| Mean canopy height + Fire disturbance history | -0.18 | -1.56 | 70 | 0.12 | [-0.40, 0.05] |
| Fire disturbance history + EVI | 0.05 | 0.45 | 70 | 0.65 | [-0.18, 0.28] |

**Table S6** - Table 1: Akaike information criterion (AIC) values for the relative contribution of all ranked models explaining the occurrence of black-fronted titi monkeys (*Callicebus nigrifrons*) in fragmented landscapes in the Cantareira-Mantiqueira Corridor.

| **Rank** | **Model** | **AICC** | **Weights** |
| --- | --- | --- | --- |
| 1 | occ3 ~ 1 + CobFlor250MB + Altitude | 67.16300 | 3.724435e-01 |
| 2 | occ3 ~ 1 + CobFlor250MB + Altitude + Fire | 69.18478 | 1.355300e-01 |
| 3 | occ3 ~ 1 + CobFlor250MB + evi + Altitude | 69.25609 | 1.307830e-01 |
| 4 | occ3 ~ 1 + CobFlor250MB + ConFun100MB + Altitude | 69.38746 | 1.224689e-01 |
| 5 | occ3 ~ 1 + CobFlor250MB + evi + Altitude + Fire | 71.39501 | 4.488405e-02 |
| 6 | occ3 ~ 1 + CobFlor250MB + ConFun100MB + Altitude + Fire | 71.49432 | 4.270975e-02 |
| 7 | occ3 ~ 1 + CobFlor250MB + ConFun100MB + evi + Altitude | 71.51325 | 4.230735e-02 |
| 8 | occ3 ~ 1 + CobFlor250MB | 72.64210 | 2.405967e-02 |
| 9 | occ3 ~ 1 + CobFlor250MB + ConFun100MB + evi + Altitude + Fire | 73.75766 | 1.377364e-02 |
| 10 | occ3 ~ 1 + CobFlor250MB + Fire | 73.77709 | 1.364049e-02 |
| 11 | occ3 ~ 1 + CobFlor250MB + evi | 73.92312 | 1.268001e-02 |
| 12 | occ3 ~ 1 + CobFlor250MB + ConFun100MB | 74.44540 | 9.765778e-03 |
| 13 | occ3 ~ 1 + CobFlor250MB + evi + Fire | 75.37019 | 6.150244e-03 |
| 14 | occ3 ~ 1 + CobFlor250MB + ConFun100MB + evi | 75.53793 | 5.655442e-03 |
| 15 | occ3 ~ 1 + ConFun100MB + evi + Altitude | 75.78620 | 4.995235e-03 |
| 16 | occ3 ~ 1 + CobFlor250MB + ConFun100MB + Fire | 75.80303 | 4.953372e-03 |
| 17 | occ3 ~ 1 + evi + Altitude | 76.15173 | 4.160856e-03 |
| 18 | occ3 ~ 1 + CobFlor250MB + ConFun100MB + evi + Fire | 77.22181 | 2.436788e-03 |
| 19 | occ3 ~ 1 + ConFun100MB + evi + Altitude + Fire | 78.09119 | 1.577736e-03 |
| 20 | occ3 ~ 1 + evi + Altitude + Fire | 78.39511 | 1.355310e-03 |
| 21 | occ3 ~ 1 + ConFun100MB + Altitude | 78.69815 | 1.164756e-03 |
| 22 | occ3 ~ 1 + Altitude | 79.25172 | 8.831359e-04 |
| 23 | occ3 ~ 1 + ConFun100MB + evi | 80.36901 | 5.051406e-04 |
| 24 | occ3 ~ 1 + ConFun100MB + Altitude + Fire | 80.93250 | 3.811106e-04 |
| 25 | occ3 ~ 1 + Altitude + Fire | 81.43064 | 2.970852e-04 |
| 26 | occ3 ~ 1 + ConFun100MB + evi + Fire | 82.05126 | 2.178291e-04 |
| 27 | occ3 ~ 1 + evi | 83.12639 | 1.272491e-04 |
| 28 | occ3 ~ 1 + evi + Fire | 84.83380 | 5.418686e-05 |
| 29 | occ3 ~ 1 + ConFun100MB | 86.54188 | 2.306691e-05 |
| 30 | occ3 ~ 1 + ConFun100MB + Fire | 88.03245 | 1.094752e-05 |
| 31 | occ3 ~ 1 | 90.68890 | 2.900528e-06 |
| 32 | occ3 ~ 1 + Fire | 92.10330 | 1.430023e-06 |

**Table S7** - Model parameter estimates

| **Parameter** | **Estimate** | **Uncond. Variance** | **Nb Models** | **Importance** | **+/- (alpha=0.05)** |
| --- | --- | --- | --- | --- | --- |
| ConFun100MB | 2.562137e-07 | 1.836442e-12 | 16 | 0.2529469 | 2.703950e-06 |
| Fire | 1.822747e-01 | 2.992285e-01 | 16 | 0.2679740 | 1.091469e+00 |
| evi | 7.948572e-01 | 5.376311e+00 | 16 | 0.2716641 | 4.626496e+00 |
| Altitude | 5.635832e-03 | 8.219568e-06 | 16 | 0.9197155 | 5.720507e-03 |
| CobFlor250MB | 4.560979e-02 | 2.294747e-04 | 16 | 0.9842420 | 3.022578e-02 |
| (Intercept) | -7.842545e+00 | 8.369656e+00 | 32 | 1.0000000 | 5.772499e+00 |
